# Comprehensive genomic signature of pyroptosis-related genes and relevant characterization in hepatocellular carcinoma

**DOI:** 10.7717/peerj.14691

**Published:** 2023-01-12

**Authors:** Sheng Wang, Songsen Gao, Liang Shan, Xueyi Qian, Jiajie Luan, Xiongwen Lv

**Affiliations:** 1 Department of Pharmacy, The First Affiliated Hospital of Wannan Medical College (Yijishan Hospital of Wannan Medical College), Wuhu, Anhui, China; 2 The Key Laboratory of Anti-Inflammatory and Immune Medicines, Ministry of Education, Anhui Province Key Laboratory of Major Autoimmune Diseases, School of Pharmacy, Institute for Liver Disease, Anhui Medical University, Hefei, Anhui, China; 3 Department of Orthopedics (Spinal Surgery), The First Affiliated Hospital of Anhui Medical University, Hefei, Anhui, China

**Keywords:** Hepatocellular carcinoma, Pyroptosis, Prognosis, Immune microenvironment, Immunotherapy

## Abstract

**Background:**

Currently, the most predominant type of liver cancer is hepatocellular carcinoma (HCC), which is also the fourth leading cause of cancer-related death in the global population. Pyroptosis is an emerging form of cell death that affects the prognosis of cancer patients by modulating tumor cell migration, proliferation and invasion. However, the evaluation of pyroptosis in the prognosis of HCC is still insufficient.

**Methods:**

A total of 365 HCC patients from the TCGA-LIHC cohort were classified into two distinct subtypes using consensus clustering of pyroptosis-related genes (PRGs). Following univariate Cox analysis of differentially expressed genes between subtypes, we established a prognostic model (PRGs-score, PRGS) by LASSO Cox analysis. We further tested the predictive power of the prognostic model in the ICGC (LIRI-JP) and GEO (GSE14520) cohorts. The tumor microenvironment (TME) was studied using the CIBERSORT. The enrichment scores for immune cells and immune functions in low- and high-PRGS groups were assessed using ssGSEA. The IMvigor210 cohort was used to investigate the immunotherapy efficacy. Furthermore, we validated the expression of prognostic genes in PRGS by RT-qPCR *in vitro*.

**Results:**

The subtyping of HCC based on PRGs exhibited distinct clinical characteristics. We developed a prognostic model PRGS by differentially expressed genes between different subtypes. The results showed that PRGS could well forecast the survival of HCC patients in different cohorts and was associated with the immune microenvironment. Moreover, PRGS was considered to be an independent prognostic risk factor and superior to other pyroptosis-related signatures. Low-PRGS implied greater immune cell infiltration and better overall survival with immunotherapy. The results of RT-qPCR also showed that prognostic genes were significantly dysregulated in HCC.

**Conclusions:**

PRGS has promising application in forecasting the prognosis of HCC patients, and its relationship with the immune microenvironment provides a basis for the subsequent treatment and research of HCC.

## Introduction

Hepatocellular carcinoma (HCC) is the most predominant primary liver cancer with increasing morbidity and mortality (Rumgay et al., 2022). Each year, more than half a million people worldwide are newly diagnosed with HCC. By 2030, HCC is predicted to cause 1 million deaths per year, thus becoming the third most lethal cancer (Bray et al., 2018; Llovet et al., 2021b; McGlynn, Petrick & El-Serag, 2021). HCC develops rapidly and insidiously, with no obvious symptoms in the early stages and a lack of effective screening tools, and many HCC patients have progressed to intermediate and advanced stages at the initial diagnosis. Currently, alpha-fetoprotein (AFP) is a generally used diagnostic and prognostic aid for HCC, but its lack of sufficient specificity and sensitivity limits its clinical application (Wang & Zhang, 2020; Zhang et al., 2020b). Moreover, HCC is a remarkably heterogeneous disease, and this heterogeneity can also affect the accuracy of prognostic prediction (Losic et al., 2020; Zhang et al., 2020a). Abnormalities in the tumor immune microenvironment have been found to be an important cause of HCC heterogeneity (Shen et al., 2020). Considering the great heterogeneity of HCC, the prediction accuracy of traditional models is still far from satisfactory, and there is a pressing need to construct a novel prognostic prediction model to enhance the specificity and sensitivity of prognosis prediction of HCC patients.

The peculiarity of pyroptosis is the cleavage of gasdermins through both classical and non-classical pathways, resulting in continuous swelling of cells until the cell membrane loses integrity, causing the release of intracellular contents, which provokes an intense inflammatory response (Kovacs & Miao, 2017; Li et al., 2021b; Liu et al., 2016; Loveless, Bloomquist & Teng, 2021). As cell death mediated by gasdermins, pyroptosis differs from other cell deaths, including necrosis and apoptosis in terms of morphological characteristics, occurrence and regulatory mechanisms (Bergsbaken, Fink & Cookson, 2009; Shi, Gao & Shao, 2017). Studies have shown that pathogens such as hepatitis virus or chemotherapy drugs can lead to the occurrence of pyroptosis in HCC (Kofahi et al., 2016; Zhang et al., 2020c). Notably, pyroptosis is closely related to the tumor immune microenvironment (Du et al., 2021; Li et al., 2021a; Xia et al., 2019). Pyroptosis can release inflammatory factors to create a tumor-inhibiting microenvironment, but it can also reduce the bodily immune response to tumor cells and promote tumorigenesis (Shao et al., 2021; Tan et al., 2021; Xia et al., 2019). Thus, pyroptosis may play a crucial role in the prognosis of cancer patients. However, the impact of pyroptosis on the prognosis of HCC patients is not well defined.

In recent years, immunotherapy has made great strides in the treatment of HCC, particularly immune checkpoint inhibitors (ICIs), which are expected to improve the treatment effect of patients (Harkus et al., 2022; Ruf, Heinrich & Greten, 2021). However, due to abnormalities in the tumor immune microenvironment, not all patients achieve the desired outcome after immunotherapy, which increases the uncertainty of patient prognosis (Labani-Motlagh, Ashja-Mahdavi & Loskog, 2020; Lin et al., 2020; Llovet et al., 2021a). Importantly, many studies have shown an intense connection between pyroptosis and cancer immunotherapy (Xiao et al., 2021; Zhao et al., 2020). Therefore, we developed a pyroptosis-related model PRGS, to forecast the prognosis of HCC patients and reveal the tumor immune microenvironment, with a view to providing guidance for immunotherapy.

In this study, we classified 365 HCC patients into distinct subtypes according to pyroptosis-related genes to develop a predictive prognostic scoring model and reveal the immune microenvironment. The two pyroptosis-related subtypes exhibited different prognostic and clinical characteristics. We developed a prognostic model PRGS using the least absolute shrinkage and selection operator (LASSO) Cox analysis of differentially expressed genes in the two subtypes. PRGS could forecast the prognosis of HCC patients from different cohorts and was intimately correlated to the immune microenvironment. Furthermore, PRGS was also an independent prognostic risk factor, and was superior to other pyroptosis-related signatures. The current study comprehensively assessed the latent link between pyroptosis, prognosis and immune microenvironment in HCC patients, providing novel ideas for improving HCC prognosis and treatment.

## Materials and Methods

### Data sources

Gene expression data, clinical characteristics and survival information of patients in three independent HCC cohorts (TCGA-LIHC, *n* = 365; LIRI-JP, *n* = 208; GSE14520, *n* = 242) were downloaded from three public databases: The Cancer Genome Atlas (TCGA, https://portal.gdc.cancer.gov/repository), the International Cancer Genomics Consortium (ICGC, Toronto, Canada, https://dcc.icgc.org/), and the Gene Expression Omnibus (GEO, https://www.ncbi.nlm.nih.gov/geo/). The criteria for data screening were to retain survival information for patients with non-zero survival time in three HCC cohorts for further analysis. Our research followed public data access policies and did not address ethical relationships. Data collection was in full accordance with the TCGA, ICGC and GEO database usage guidelines. Furthermore, an immunotherapy dataset (Imvigor210) was downloaded using the “IMvigor210CoreBiologies” R package (Mariathasan et al., 2018). Meanwhile, the 48 PRGs used in this study were extracted from the MsigDB database (http://www.gsea-msigdb.org/gsea/msigdb/) and published literature (**Table S1**) (Shao et al., 2021; Ye, Dai & Qi, 2021). The flow chart of our work is illustrated in Fig. S1.

### Consensus clustering analysis of differentially expressed PRGs

Differentially expressed genes (DEGs) in 365 HCC samples and 50 adjacent non-tumor samples from the TCGA-LIHC cohort were determined using the “limma” R package with the criteria of |log2(FC)| > log2(1.5) and FDR < 0.05, and then differentially expressed PRGs were extracted from DEGs. The volcano plot of DEGs was drawn using the “ggplot2” R package.

Consensus unsupervised clustering analysis was implemented using the “Consensus ClusterPlus” R package to divide HCC patients into distinct subtypes according to differentially expressed PRGs. Consensus clustering was performed using the k-means method and 1,000 repetitions were run to ensure the stability of the classification. This clustering met the following requirements: the cumulative distribution function (CDF) curve slowly and gradually elevated; the number of patients without any group was small; after clustering, the intergroup and intragroup correlation decreased and increased, respectively. Meanwhile, the optimum k value for consensus clustering was determined by combining the proportion of ambiguous clustering (PAC). The heatmap plot of PRGs was drawn using the “pheatmap” R package. The difference in overall survival (OS) between the two subtypes was analyzed using Kaplan–Meier curves.

### Determination and functional annotation of DEGs between the two subtypes

DEGs in the two pyroptosis-related subtypes were determined by the “limma” R package based on the following criteria: |log2(FC)| > 2 and FDR < 0.01. We performed Kyoto Encyclopedia of Genes and Genomes (KEGG) as well as Gene Ontology (GO) functional enrichment analysis of pyroptosis-related DEGs based on the “clusterprofiler” R package to investigate the latent functions of these DEGs and identify relevant enrichment pathways. Following, pathways and terms were defined as statistically significant using FDR < 0.05.

### Construction of the pyroptosis-related prognostic PRGS

We utilized univariate Cox regression analysis to determine the pyroptosis-related DEGs correlated with OS in the training cohort (TCGA-LIHC, *n* = 365), screened by the criterion of *p*-value < 0.0001. Next, the LASSO Cox algorithm eliminated overfitting between prognostic genes to perform penalty parameter tuning by ten-fold cross validation according to the “glmnet” R package in an attempt to narrow the range of prognostic genes. The pyroptosis-related risk score for each patient with HCC was calculated using gene expression levels multiplied by the LASSO Cox regression coefficients. Based on this, we constructed an efficient prognostic prediction model PRGS to obtain the optimal prediction outcomes from the TCGA-LIHC cohort using overall survival.

The calculation formula of PRGS was: PRGS = Σ (Coefi * Expi), where Coefi and Expi denote the regression coefficient and corresponding expression level of each prognostic gene, respectively. A total of 365 patients from the TCGA-LIHC cohort were classified into low-risk (PRGS < median value) and high-risk (PRGS > median value) groups according to the median PRGS, followed by Kaplan–Meier survival and receiver operating characteristic (ROC) curves to assess the predictive power of PRGS model. Then, we performed principal component analysis (PCA) according to the “factoextra” R package. Likewise, HCC patients in the testing cohorts (LIRI-JP and GSE14520) were also classified into low- and high-risk groups, and each cohort underwent Kaplan–Meier survival and ROC curves.

### Clinical correlation analysis of the prognostic PRGS

The Chi-square test was performed to assess the correlation between PRGS and multiple clinical characteristics (age, gender, stage, TNM, status, grade). Univariate and multivariate Cox regression analyses were conducted in the training and testing cohorts to assess whether PRGS was a prognostic factor independent of other available clinical characteristics. The *p*-value < 0.05 for pyroptosis-related signature PRGS and other clinical characteristics were considered as independent prognostic risk factors.

### Development and validation of the nomogram

We developed a nomogram model to forecast the survival time of HCC patients according to the prognostic model PRGS and different clinical characteristics (age, gender, stage,grade, and T stage) using the “survival” and “rms” R packages. In the nomogram, each factor has a corresponding score, and the scores of all factors for every patient are added together to gain the total score to predict the corresponding overall survival time. The C-index was applied to appraise the discriminative ability of the nomogram. The predictive power of the nomogram was assessed using ROC curves for 1-, 3-, and 4-year survival. Furthermore, we used calibration plots of the nomogram to exhibit the consistency between the actual observed survival and the predicted 1-, 3-, and 4-year survival.

### Evaluation of the tumor immune microenvironment

To assess the proportions of infiltrating immune cells in the tumor immune microenvironment, we utilized the CIBERSORT algorithm to calculate the abundance of 22 infiltrating immune cells in low- and high-risk groups. Next, we applied single-sample gene set enrichment analysis (ssGSEA), the enrichment scores for 13 immune functions and 16 immune cells in HCC patients were calculated based on the specific expressed gene signatures using the “GSVA” R package. In addition, the associations between PRGS and the enrichment scores for 13 immune functions and 16 immune cells were also investigated.

### Immunotherapy efficacy and drug susceptibility analysis

Immunophenoscore (IPS) is a good predictor of ICIs response, and its score is determined by quantifying vital components of tumor immunity. Specifically, IPS scores are calculated based on z-scores of immune-related gene expression in representative cell types, ranging from 0 to 10. High IPS scores reflect enhanced immunogenicity. We obtained IPS scores for patients with HCC (from the TCGA-LIHC cohort) from The Cancer Immunome Atlas (TCIA, https://www.tcia.at/home), and compared the distribution of IPS scores in low- and high-risk groups. Additionally, the expression of several typical immune checkpoints in low- and high-risk groups was also analyzed. The IMvigor210 cohort was applied to assess the immunotherapy efficacy in low- and high-risk groups.

To assess differences in the efficacy of targeted therapy and chemotherapy drugs between patients in low- and high-risk groups, we applied the “pRRophetic” R package to predict the half-maximal inhibitory concentrations (IC50) values of chemotherapeutic drugs commonly utilized to treat HCC. The common chemotherapeutic drugs, such as cisplatin, gefitinib, sorafenib were selected for HCC.

### Cell culture and real-time PCR analysis

Human normal liver LO2 cell line and liver cancer Huh7 and HepG2 cell lines (obtained from the cell bank of the Chinese Academy of Science) were applied to detect the expression of five prognostic genes of PRGS. Total RNA from LO2, Huh7 and HepG2 cell lines was isolated using Trizol reagent. Total RNA was pretreated with RNase‑free DNase and then reverse‑transcribed with AMV to synthesize cDNA. The RT-qPCR reactions were conducted using the Light Cycler 480 SYBR Green I kit with specific primers commercially synthesized by Invitrogen Biotechnology Ltd. (Table S2). The 20 μl of reaction mixture was composed of 1 μl of diluted cDNA, 2 μl of primer mix (reverse and forward primers), 7 μl of 10X buffer and 10 μl of Mix (2X PCR Buffer, 2X dNTP, 2X Taq DNA Polymerase). We performed amplification reactions using the LightCycler® 480 instrument with an initial step at 95 °C for 5 min, followed by 50 cycles of three step PCR at 95 °C for 15 s, 60 °C for 15 s and 72 °C for 30 s. Quantitative analysis was normalized using β-actin.

### Statistical analysis

The Chi-square test and Student’s t-tests were utilized to calculate the significant differences between variables. For baseline clinical data, Kruskal–Wallis and Wilcoxon rank-sum tests were implemented to assess statistical differences between multiple and two groups, respectively. The log-rank test was utilized to assess differences in Kaplan–Meier curves. The *p*-value < 0.05 represented a statistically significant difference. All statistical analyses involving data in this study were conducted using R 4.0.3 and Prism 8 software.

## Results

### Genetic and expression alterations of PRGs in HCC

**Figure 1:**
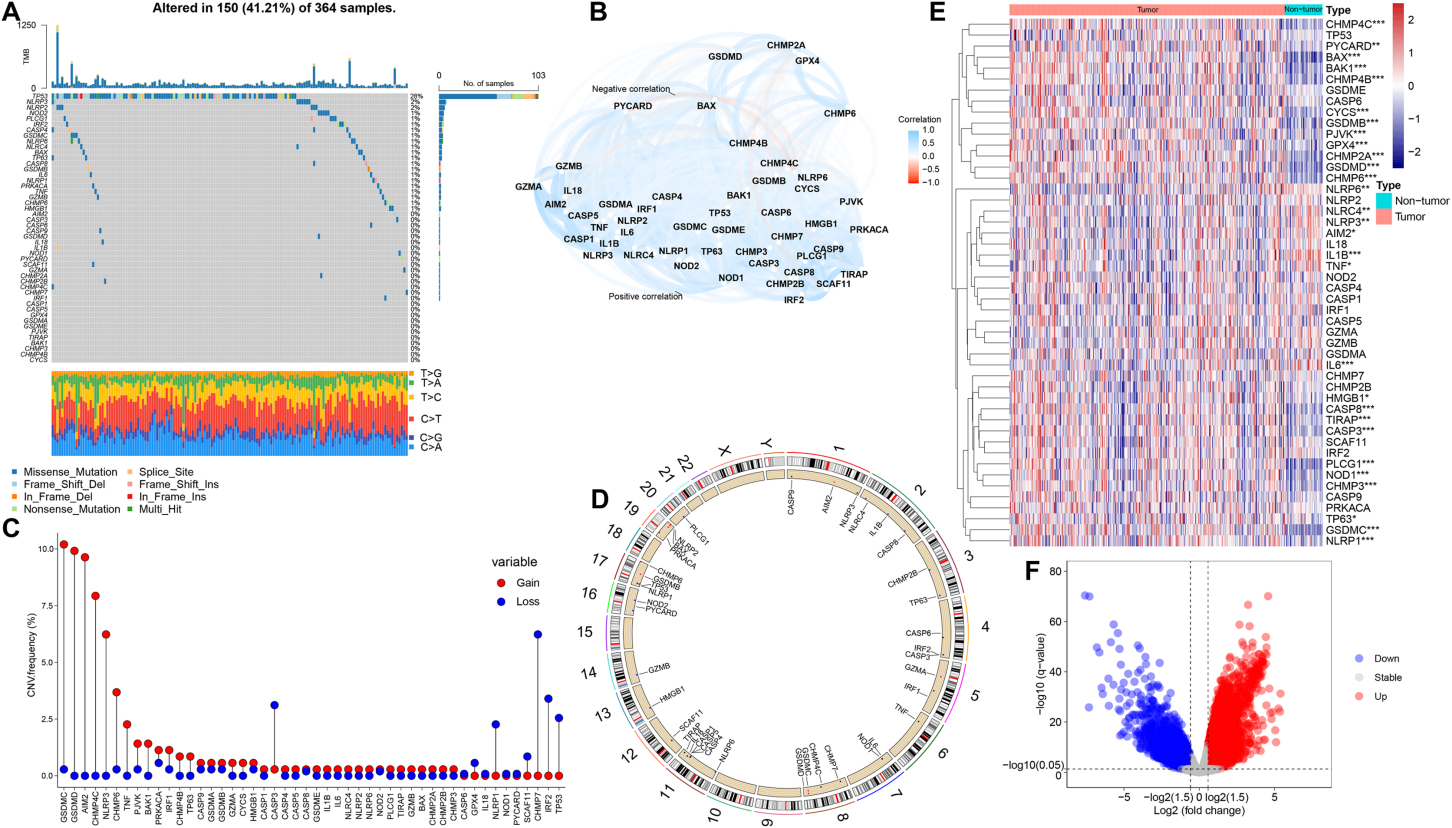
Genetic and expression alterations of PRGs in HCC. (A) Mutation incidence of 48 PRGs in 364 HCC patients in the TCGA-LIHC cohort. (B) The correlation network of 48 PRGs (redline: negative correlation; blueline: positive correlation). (C) CNV frequencies of PRGs in the TCGA-LIHC cohort (the height of the columns revealed different proportions of CNV gain and loss). (D) The location of CNV alterations in PRGs on their corresponding chromosomes. (E) Differential expression distribution of 48 PRGs between HCC and non-tumor samples. (F) Volcano plot of DEGs between HCC and adjacent non-tumor samples. *p < 0.05; **p < 0.01; ***p < 0.001.

In this study, we selected 48 PRGs for analysis. At the genetic level, the occurrence of somatic mutations in 48 PRGs was analyzed, and the results showed that 150 (41.21%) of the 364 HCC samples had mutations in the PRGs (Fig. 1A). Among all 48 PRGs, the gene with the highest mutation incidence was TP53 (28%), followed by NLRP3 and NLRP2. Figure 1B depicts the correlation network including all PRGs. We then evaluated somatic copy number alterations in 48 PRGs and found that such alterations were present in most PRGs. Among these PRGs, copy number variation (CNV) of GSDMC, GSDMD, AIM2, CHMP4C, NLRP3 and CHMP6 was generally increased, while those of CASP3, NLRP1, CHMP7 and IRF2 was decreased (Fig. 1C). Figure 1D exhibits the location of CNV alterations in PRGs on their corresponding chromosomes.

To compare the differential expression of PRGs in 365 HCC samples and 50 non-tumor samples from the training (TCGA-LIHC) cohort, we first conducted differential expression analysis on all genes, and then identified the corresponding expression differences for 48 PRGs (Fig. 1E). Volcano plot was utilized to describe the DEGs between HCC and non-tumor samples (Fig. 1F). We further analyzed the possible relationship between CNV alterations and differential expression of PRGs. Interestingly, PRGs with CNV gain, including GSDMD, GSDMC, CHMP4C and CHMP6, were significantly higher in HCC samples than in adjacent non-tumor samples (*p* < 0.001), suggesting that CNVs may regulate PRGs expression. However, compared with non-tumor samples, some PRGs with CNV gain, including NLRP3, were significantly downregulated in HCC (*p* < 0.01); some PRGs with CNV loss, including CASP3, were significantly upregulated in HCC (*p* < 0.001). Therefore, although CNV can be used to explain most of the alterations in PRGs expression, CNV is not a determinant of gene expression regulation (Stranger et al., 2007). Other important factors modulating gene expression levels include histone modifications and DNA methylation (Angeloni & Bogdanovic, 2019; Pope & Medzhitov, 2018). Our analysis revealed remarkable differences in genetic alterations and expression levels of PRGs in HCC and non-tumor samples, suggesting a latent role of PRGs in HCC tumorigenesis.

### Identification of pyroptosis-related subtypes in HCC

**Figure 2:**
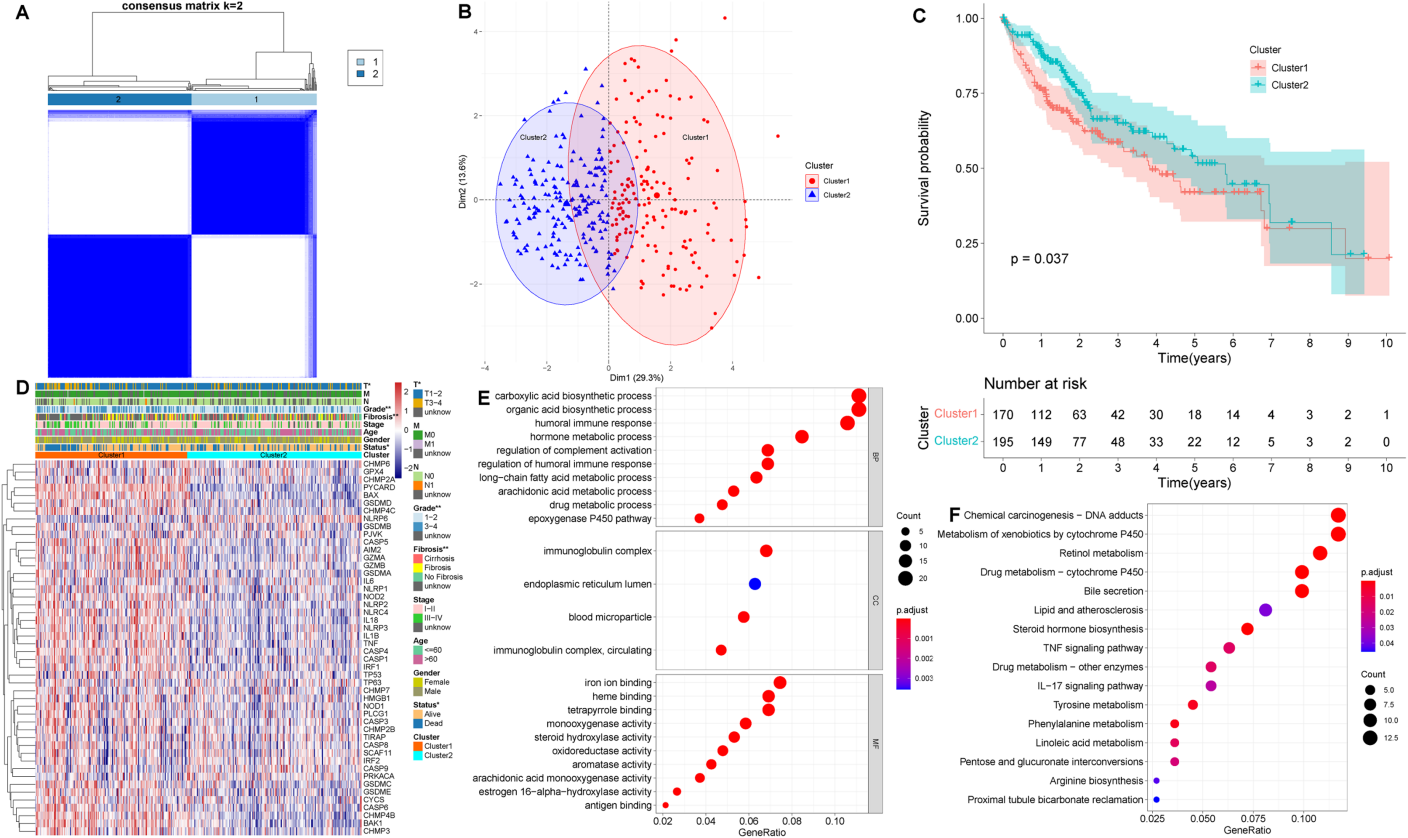
Clinical and biological characteristics of pyroptosis-related subtypes in HCC. (A) The HCC patients were classified into two clusters based on the consensus clustering variable k = 2. When two samples had higher consensus score in different iterations, they were more likely to be clustered together. (B) PCA plot showing that cluster 1 and cluster 2 were clustered separately. (C) Kaplan–Meier curves of OS in HCC patients with two pyroptosis-related subtypes from the TCGA-LIHC cohort. (D) Differences in clinical characteristics and expression levels of PRGs between cluster 1 and cluster 2. (E, F) GO and KEGG analysis of pyroptosis-related DEGs between cluster 1 and cluster 2. *p < 0.05; **p < 0.01.

Based on the levels of differentially expressed PRGs, we identified distinct pyroptosis-related subtypes in the training (TCGA-LIHC) cohort using a consensus clustering algorithm (Fig. S2). Our results suggested that the clustering variable k = 2 seemed to be the optimum choice for classifying the whole cohort into pyroptosis-related cluster 1 (*n* = 170) and pyroptosis-related cluster 2 (*n* = 195) (Fig. 2A**, Table S3**). The results of principal component analysis (PCA) suggested that two pyroptosis-related subtypes, namely cluster 1 and cluster 2, were clustered separately (Fig. 2B). Besides, Silhouette analysis showed results consistent with PCA (Fig. S3). Kaplan–Meier curves showed that patients in cluster 2 had significantly longer OS than those in cluster 1 (*p* = 0.037, Fig. 2C). In addition, comparing the clinical characteristics of cluster 1 and cluster 2 in HCC using the Chi-square test revealed remarkable differences between the two subtypes. As shown in Fig. 2D, cluster 2 had better survival status, lower grade, and lower T stage than cluster 1 (*p* < 0.05), which corresponds to the previously described cluster 2 having longer OS.

Next, we identified DEGs between two pyroptosis-related subtypes, and used the obtained pyroptosis-related DEGs for subsequent analysis. To evaluate the latent functions of pyroptosis-related subtypes, we identified 203 pyroptosis-related DEGs in cluster 1 and cluster 2, and then conducted biological function analysis, including GO and KEGG enrichment analysis (Figs. 2E and 2F). According to GO analysis, in biological process (BP) category, pyroptosis-related DEGs were primarily enriched in drug metabolic process, humoral immune response and regulation of complement activation; in cellular component (CC) term, the pyroptosis-related DEGs were primarily enriched in blood microparticle, endoplasmic reticulum lumen and immunoglobulin complex; regarding molecular function (MF), pyroptosis-related DEGs were primarily enriched in antigen binding and iron ion binding (Fig. 2E). As for KEGG pathway analysis, pyroptosis-related DEGs were significantly enriched in chemical carcinogenesis, TNF signaling pathway and IL-17 signaling pathway (Fig. 2F).

### Construction and validation of the prognostic PRGS

**Figure 3:**
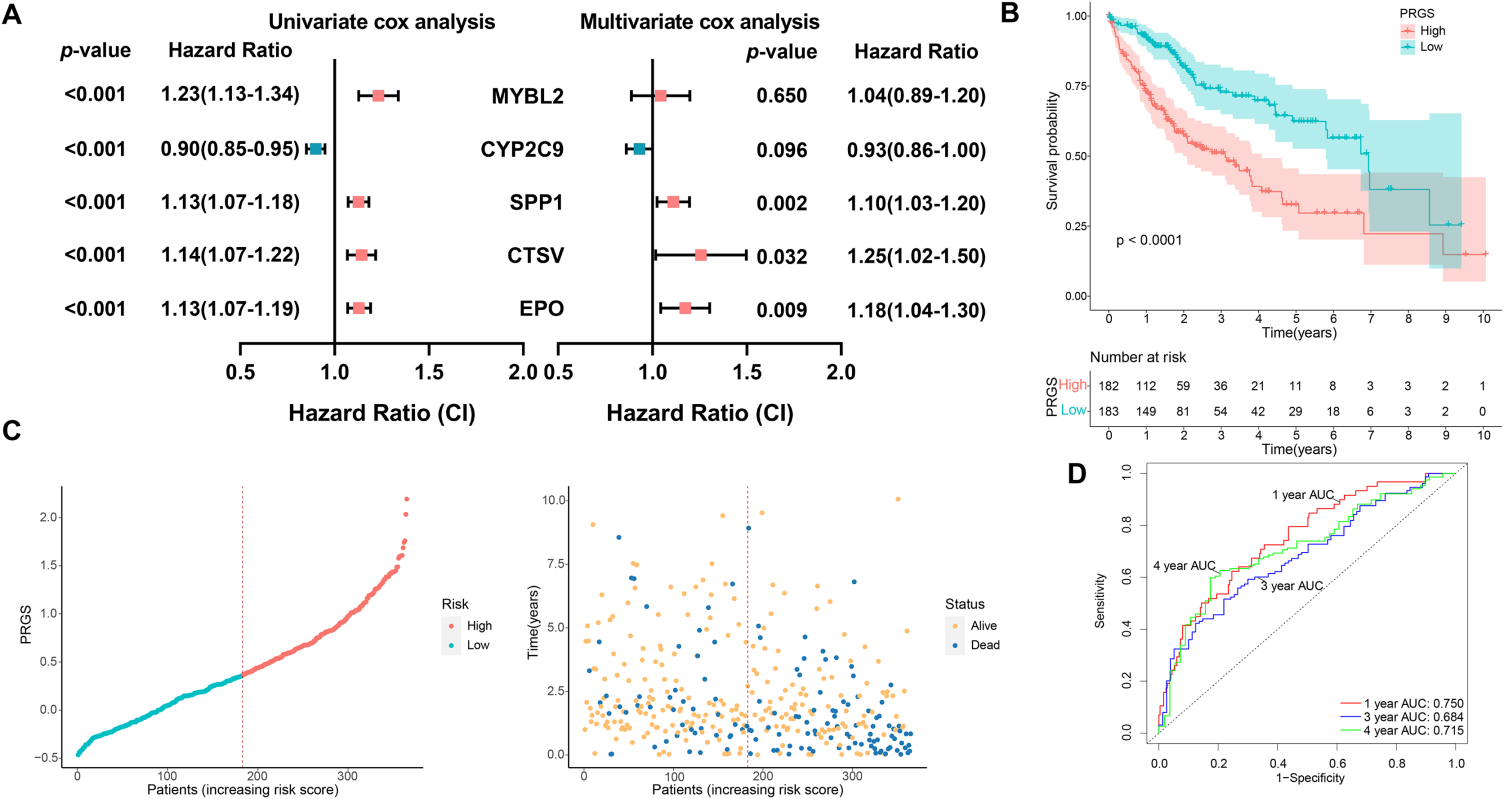
Construction of the prognostic PRGS in the TCGA-LIHC cohort. (A) Forest plot demonstrating univariate and multivariate Cox results of five prognostic genes in PRGS. (B) Kaplan–Meier curves of OS in the TCGA-LIHC cohort divided into low- and high-risk groups. (C) Risk score distribution of the TCGA-LIHC cohort, and survival status of low- and high-risk groups. (D) ROC curve of sensitivity and specificity for predicting 1-, 3-, and 4-year survival based on PRGS in the TCGA-LIHC cohort.

A total of 365 HCC patients in the TCGA-LIHC cohort were utilized to construct the prognostic prediction model. First, univariate Cox analysis was performed to determine the association between 203 pyroptosis-related DEGs and OS outcomes in HCC patients. Among the 203 pyroptosis-related DEGs, five genes were screened. To avoid overfitting between genes, we performed LASSO Cox regression using a minimized lambda (Fig. S4A). The results of the LASSO Cox analysis suggested that all five genes had non-zero regression coefficients and were extracted for subsequent analysis. These five genes and their corresponding coefficients were utilized to develop a pyroptosis-related gene scoring model, named “PRGS”. These five prognostic genes were utilized to construct the model PRGS, and the calculation formula was as follows: Risk score = (−0.06198467 * expression level of CYP2C9) + (0.03381356 * expression level of MYBL2) + (0.08247219 * expression level of SPP1) + (0.20457157 * expression level of CTSV) + (0.15033404 * expression level of EPO) (Fig. 3A).

Based on the above calculation formula, we calculated the risk score for every patient in the TCGA-LIHC cohort. Then, based on the median PRGS, patients were classified into low- and high-risk groups. The results of PCA showed that low- and high-risk groups were clustered separately (Fig. S4B). The overall survival of patients in the low-risk group was significantly better than that in the high-risk group (*p* < 0.0001, Fig. 3B). The distribution of PRGS, survival time and survival status for low- and high-risk groups were shown in Fig. 3C, and patients with death status increased with PRGS. As exhibited in Fig. 3D, in the training cohort, the AUC values of PRGS for 1-, 3-, and 4-year were 0.750, 0.684, and 0.715, respectively. We performed univariate and multivariate Cox regression analyses to investigate in depth whether PRGS model was independent of other clinical characteristics, including age, gender, grading, T stage and stage (Table 1). The results indicated that PRGS was identified as an independent prognostic risk factor (HR = 2.33, 95% CI [1.55–3.50], *p* < 0.001).

**Table 1:** Univariate and multivariate Cox regression analyses of PRGS and clinical characteristics in three cohorts.

Variables	Univariate analysis				Multivariate analysis	
	HR	95% CI	*p*-value	HR	95% CI	*p*-value
TCGA-LIHC						
Age (>60 *vs*. <=60)	1.29	[0.89–1.87]	0.174	1.25	[0.86–1.80]	0.251
Gender (male *vs*. female)	0.78	[0.54–1.14]	0.203	0.94	[0.64–1.40]	0.751
Stage (III–IV *vs*. I–II)	2.45	[1.69–3.56]	<0.001***	0.88	[0.12–6.50]	0.897
Grade (3–4 *vs*. 1–2)	1.16	[0.80–1.69]	0.427	1.03	[0.70–1.50]	0.874
T (T3-4 *vs*. T1-2)	2.48	[1.70–3.60]	<0.001***	2.61	[0.35–19.40]	0.348
PRGS (high-risk *vs*. low-risk)	2.57	[1.74–3.80]	<0.001***	2.33	[1.55–3.50]	<0.001***
LIRI-JP						
Age (>60 *vs*. <=60)	0.81	[0.39–1.67]	0.572	0.67	[0.32–1.43]	0.301
Gender (male *vs*. female)	0.47	[0.24–0.91]	0.026*	0.37	[0.18–0.76]	0.007**
Stage (III–IV *vs*. I–II)	2.95	[1.52–5.72]	0.001**	3.19	[1.60– 6.34]	<0.001***
PRGS (high-risk *vs*. low-risk)	4.14	[1.96–8.77]	<0.001***	3.48	[1.63–7.43]	0.001**
GSE14520						
Gender (male *vs*. female)	1.63	[0.79–3.38]	0.188	1.22	[0.58–2.60]	0.601
Age (>60 *vs*. <=60)	0.93	[0.53–1.62]	0.788	1.05	[0.58–1.90]	0.879
HBV (Carrier *vs*. Normal)	1.15	[0.28–4.68]	0.845	1.53	[0.36–6.50]	0.564
ALT (>50 U/L *vs*. <=50 U/L)	1.10	[0.71–1.68]	0.673	0.86	[0.54–1.40]	0.511
Tumor size (>5 cm *vs*. <=5 cm)	2.16	[1.41–3.32]	<0.001***	0.92	[0.51–1.70]	0.787
Multinodular (Yes *vs*. No)	1.54	[0.96–2.47]	0.074	0.49	[0.23–1.10]	0.070
Cirrhosis (Yes *vs*. No)	4.67	[1.15–18.97]	0.031*	3.83	[0.91–16.00]	0.066
TNM (III–IV *vs*. I–II)	3.47	[2.22–5.41]	<0.001***	1.41	[0.64–3.10]	0.397
BCLC (B *vs*. A)	2.46	[1.33–2.88]	0.004**	3.27	[1.16–9.20]	0.025*
BCLC (C *vs*. A)	4.83	[4.54–8.1]	<0.001***	4.97	[2.17–11.40]	<0.001***
AFP (>300 ng/ml *vs*. <=300 ng/ml)	1.61	[1.05–2.46]	0.029*	1.27	[0.82–2.00]	0.288
PRGS (high-risk *vs*. low-risk)	2.01	[1.31–3.11]	0.002**	1.61	[1.01–2.60]	0.047*

**Notes:**

* *p* < 0.05.

** *p* < 0.01.

*** *p* < 0.001.

**Figure 4:**
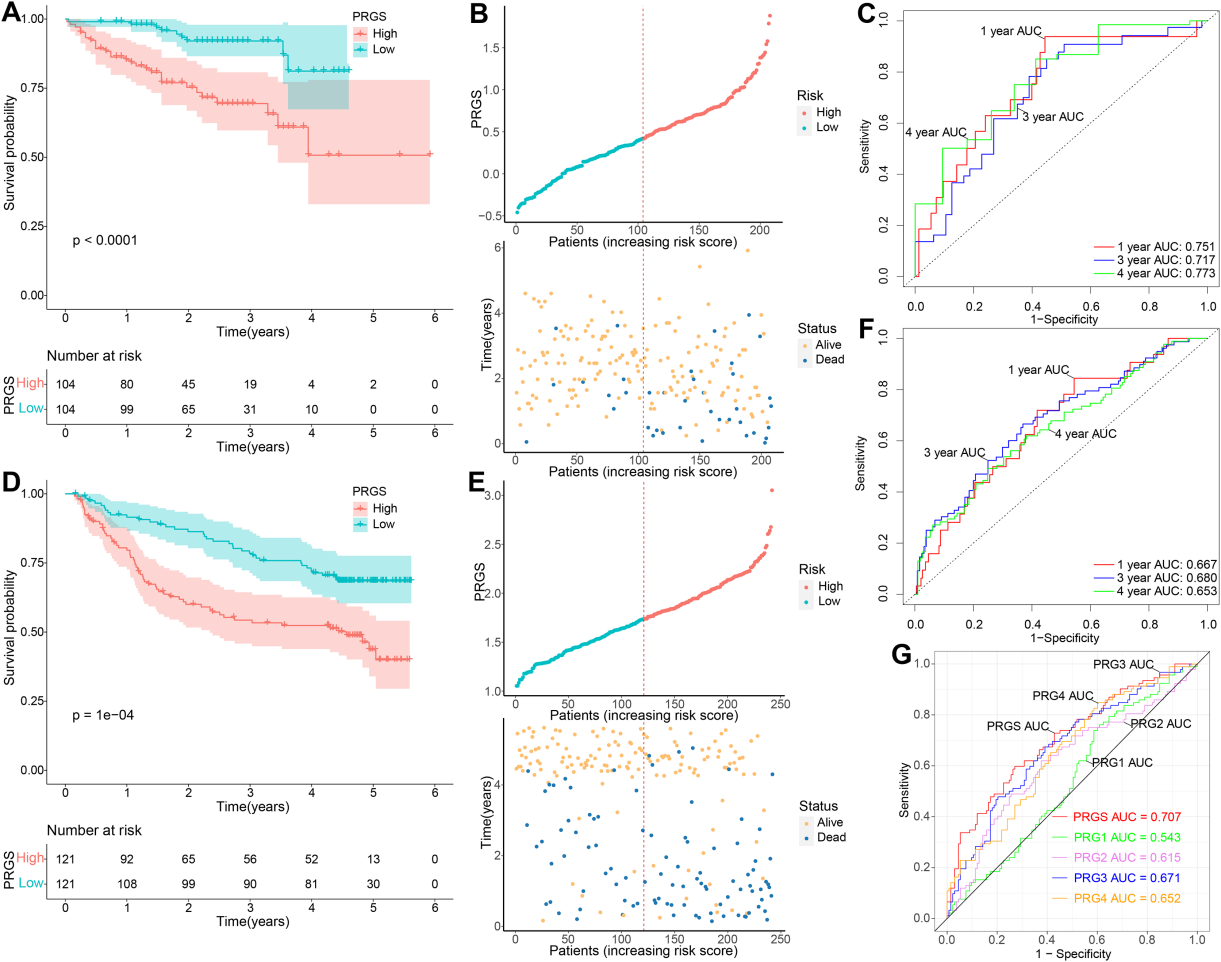
Validation of the prognostic PRGS in the testing cohorts. (A) Kaplan–Meier curves of overall survival in the LIRI-JP cohort grouped into low- and high-risk groups. (B) Risk score distribution of the LIRI-JP cohort, and survival status of low- and high-risk groups. (C) ROC curve of sensitivity and specificity for predicting 1-, 3-, and 4-year survival according to PRGS in the LIRI-JP cohort. (D) Kaplan–Meier curves of overall survival in the GSE14520 cohort divided into low- and high-risk groups. (E) Risk score distribution of the GSE14520 cohort, and survival status of low- and high-risk groups. (F) ROC curves of sensitivity and specificity for predicting 1-, 3-, and 4-year survival based on PRGS in the GSE14520 cohort. (G) ROC curves for the predictive power of PRGS and other published pyroptosis-related signatures.

To demonstrate the robustness and versatility of model PRGS, we also validated this risk score in the testing cohorts (LIRI-JP and GSE14520) and obtained similar results. We used the same formula to classify patients in the testing cohorts into low- and high-risk groups. In the LIRI-JP cohort, the OS of patients in the low-risk group was significantly prolonged compared to patients in the high-risk group (*p* < 0.0001, Fig. 4A). The distribution of PRGS, survival time and survival status were exhibited in Fig. 4B, and patients with death status increased with PRGS. The AUC values of PRGS for 1-, 3-, and 4-year were 0.751, 0.717, and 0.773, respectively (Fig. 4C). Likewise, in the GSE14520 cohort, patients in the low-risk group also had significantly longer overall survival than those in the high-risk group (*p* = 0.0001, Fig. 4D). The distribution of PRGS, survival time and survival status were shown in Fig. 4E, and patients with survival status decreased with increasing PRGS. The AUC values of PRGS for 1-, 3-, and 4-year were 0.667, 0.680, and 0.653, respectively (Fig. 4F). Similarly, univariate and multivariate Cox regression analyses of PRGS and clinical characteristics such as age, gender and stage were carried out in the testing cohorts (Table 1). As a result, PRGS was an independent prognostic factor with excellent predictive power for survival in HCC patients (HR = 3.48, 95% CI [1.63–7.43], *p* = 0.001; HR = 1.61, 95% CI [1.01–2.60], *p* = 0.047). Moreover, in the training and testing cohorts, we applied ROC curves to compare the prognostic predictive power of PRGS with other pyroptosis-related signatures. We found that PRGS consistently maintained superior performance compared to other published pyroptosis-related signatures (Figs. 4G and S5, **Table S4**) (Fu & Song, 2021; Jin et al., 2022; Liu et al., 2021; Wu et al., 2021b). These results suggested that PRGS was an extremely reliable prognostic prediction model and outperformed other pyroptosis-related signatures.

### Validation of the expression levels of five genes for PRGS

**Figure 5:**
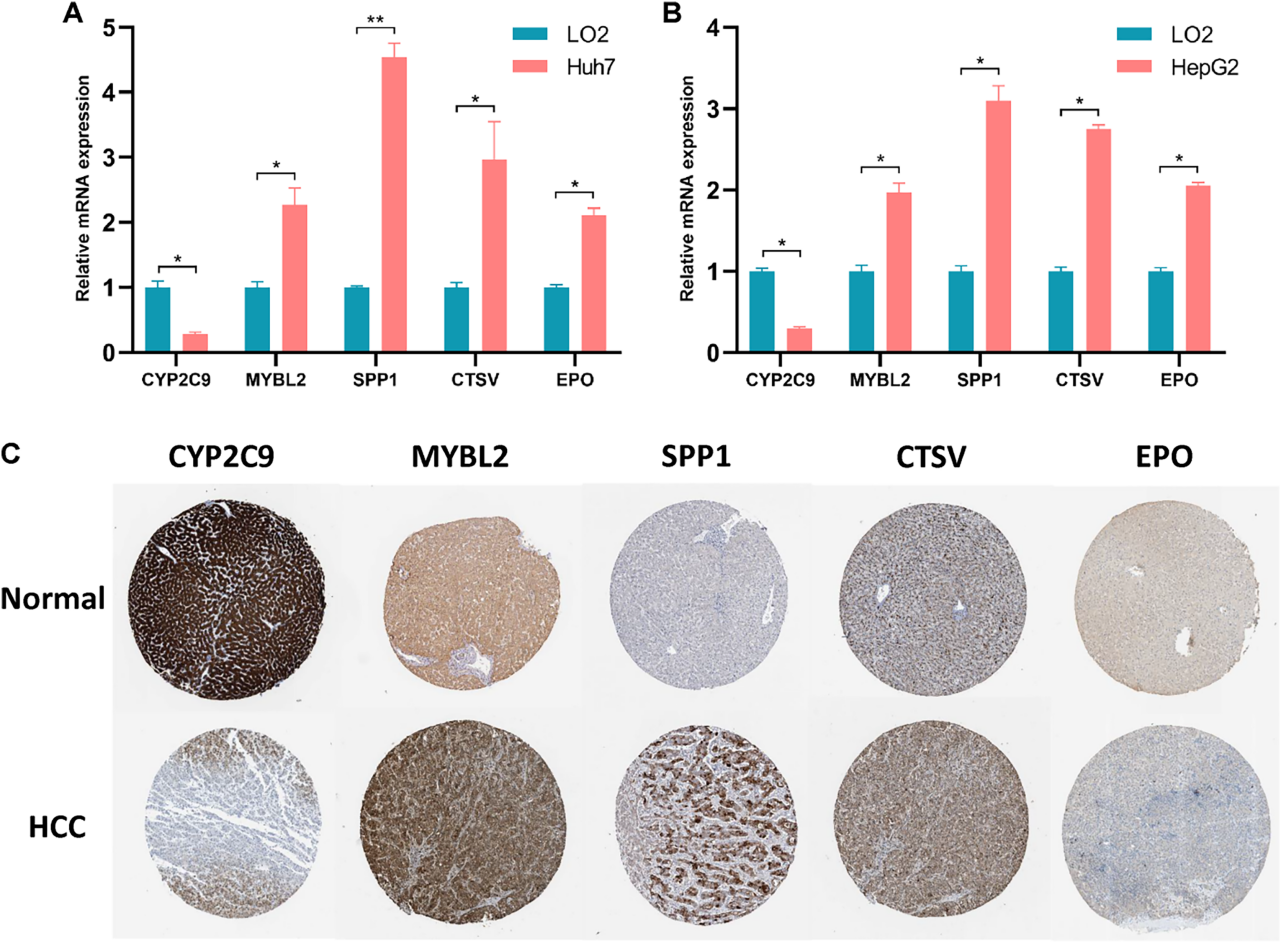
Validation of the expression levels of 5 prognostic genes in PRGS. (A, B) Expression of five prognostic genes (CYP2C9, MYBL2, SPP1, CTSV and EPO) constituting the model in LO2, Huh7 and HepG2 cell lines. (C) IHC staining plot showing representative images of protein expression levels of five prognostic genes in normal and HCC liver tissues obtained from the HPA database. *p < 0.05; **p < 0.01.

The expression levels of five prognostic genes in human normal liver cells and liver cancer cells were detected by RT-qPCR. As illustrated in Figs. 5A and 5B, the expression levels of MYBL2, SPP1, CTSV and EPO were remarkably increased in liver cancer cells compared with normal liver cells, while the expression level of CYP2C9 was significantly decreased (*p* < 0.05). Notably, the expression of these five genes was not statistically different between Huh7 and HepG2 cell lines (Fig. S6). Furthermore, we assessed the protein levels of these five prognostic genes in normal and HCC liver tissues using the Human Protein Atlas (HPA, https://www.proteinatlas.org/) database. Immunohistochemical (IHC) staining information for five prognostic genes in normal and HCC liver tissues from the HPA database was reflected in Fig. 5C. We found that the protein levels of SPP1, MYBL2, CTSV and EPO were increased in HCC tissues, while the protein level of CYP2C9 was decreased.

### Associations between PRGS and clinical characteristics of HCC patients

**Figure 6:**
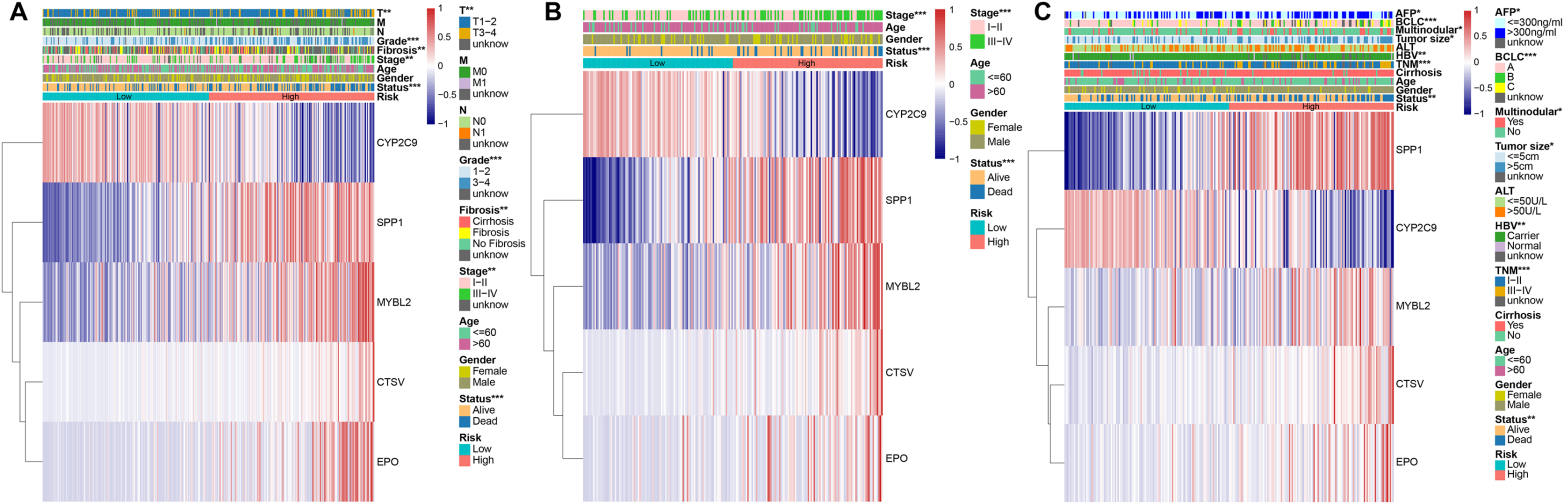
Associations between PRGS and clinical characteristics of HCC patients. (A) Relationships between clinical characteristics and low- and high-risk groups in TCGA-LIHC cohort. (B) Relationships between clinical characteristics and low- and high-risk groups in LIRI-JP cohort. (C) Relationships between clinical characteristics and low- and high-risk groups in GSE14520 cohort. *p < 0.05; **p < 0.01; ***p < 0.001.

To evaluate the association between PRGS and clinical characteristics, we explored the clinical manifestations of low- and high-risk groups in both the training and testing cohorts, including age, gender, stage, TNM, status, grade. We created heatmaps of clinical characteristics for three cohorts (Figs. 6A–6C). In three cohorts, except for CYP2C9, the expression levels of the remaining of the four genes (MYBL2, SPP1, CTSV and EPO) were elevated in the in high-risk group. We observed that the survival status of HCC patients in three cohorts was extremely consistent with the overall survival advantage described in our previous study (Figs. 6A–6C). In the TCGA-LIHC and LIRI-JP cohorts, early stage (I–II) patients dominated the low-risk group, whereas advanced stage (III–IV) patients were mainly divided into the high-risk group (*p* < 0.01, Figs. 6A and 6B). Likewise, in the TCGA-LIHC and GSE14520 cohorts, patients with early T (or TNM) stage were predominantly distributed in the low-risk group, whereas patients with advanced T (or TNM) were mostly distributed in the high-risk group (*p* < 0.01, Figs. 6A, 6C). This explains why the low-risk group was associated with a better overall survival advantage. Importantly, in the GSE14520 cohort, we noted that AFP (<=300 ng/ml), tumor size (<=5 cm) and non-multinodular were associated with the low-risk group, whereas AFP (>300 ng/ml), tumor size (>5 cm) and multinodular were mainly associated with the high-risk group (*p* < 0.05, Fig. 6C). In addition, in the TCGA-LIHC cohort, we also assessed whether PRGS maintained its predictive power in various clinical characteristic subgroups, such as age (<=60 and >60 years), gender (female and male), stage (I–II and III–IV), grade (1–2 and 3–4), T stage (T1-2 and T3-4), and fibrosis (fibrosis & cirrhosis and no fibrosis), and the results showed that PRGS could be used to forecast OS in various subgroups of clinical characteristics (except for the female subgroup) (*p* < 0.05, Fig. S7). These findings illustrated that low- and high-risk groups of PRGS represented distinct clinical characteristics of HCC patients, and that PRGS could maintain excellent predictive power in clinical characteristic subgroups.

### Establishment and evaluation of the predictive nomogram

**Figure 7:**
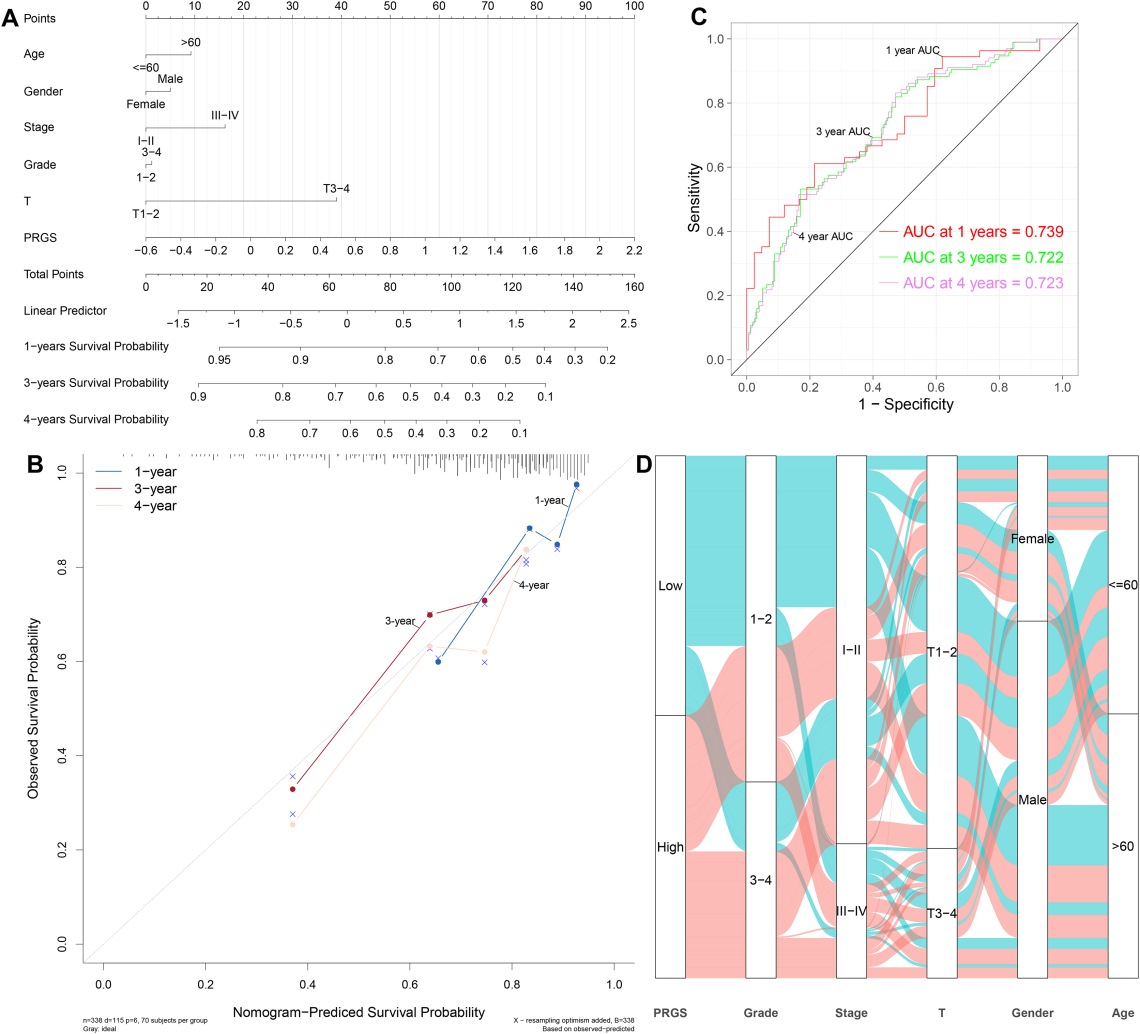
Establishment and evaluation of the nomogram to predict the OS in HCC patients. (A) Nomogram containing PRGS to predict the overall survival in the TCGA-LIHC cohort. (B) Calibration plot of the nomogram on consistency between predicted and observed 1-, 3-, and 4-year survival in the TCGA-LIHC cohort. (C) Time-dependent ROC curves for predicting 1-, 3-, and 4-year survival using the nomogram. (D) Alluvial diagram showing the changes in PRGS, grade, stage, T stage, gender and age in the TCGA-LIHC cohort.

Considering the importance of PRGS in the prognosis prediction of HCC patients, we then tried to investigate its feasibility in clinical applications. We established a nomogram containing PRGS and clinical characteristics that were readily available and recognized to have some degree of influence on the prognosis of HCC, to predict 1-, 3-, and 4-year survival in the TCGA-LIHC cohort (Fig. 7A). The C-index of 0.715 demonstrated the excellent predictive power of the nomogram. Calibration plot of the nomogram indicated strong agreement between observed and predicted 1-, 3-, and 4-year survival (Fig. 7B). The AUC values of the nomogram model at 3-, and 4-year showed higher accuracy in predicting survival (Fig. 7C). Specifically, the AUC values of the nomogram for 1-, 3-, and 4-year were 0.739, 0.722, and 0.723, respectively. As shown in Fig. 7D, the alluvial plot was utilized to describe changes in the characteristics of HCC patients.

Furthermore, we also constructed nomograms in the LIRI-JP and GSE14520 cohorts to forecast 1-, 3-, and 4-year survival (Figs. S8A and S8B). The C-index of the nomogram was 0.769 in the LIRI-JP cohort and 0.702 in the GSE14520 cohort, indicating superior predictive performance for both nomograms. Calibration plots showed that the nomograms could accurately estimate 1-, 3-, and 4-year survival (Figs. S8C and S8D). The 1-, 3-, and 4-year AUC values of the nomograms in the LIRI-JP and GSE14520 cohorts were 0.726, 0.758, and 0.744, as well as 0.728, 0.726, and 0.599, showing good accuracy (Figs. S8E and S8F). In addition, in the training and testing cohorts, we compared the prognostic predictive power of our nomogram with other published pyroptosis-related nomograms (Fu & Song, 2021; Jin et al., 2022; Liu et al., 2021). The results showed that our nomogram has better predictive power (Figs. S8G–S8I, **Table S5**). According to the above results, we observed that PRGS can play a critical role in the clinical prognosis prediction of HCC patients.

### Evaluation of the immune microenvironment between low- and high-risk groups

**Figure 8:**
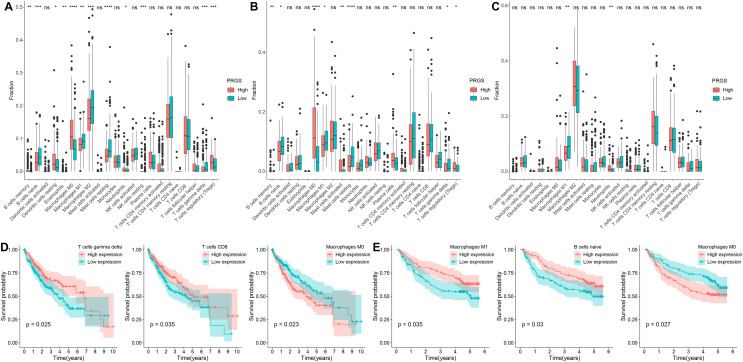
Analysis of the immune microenvironment in low- and high-risk groups. (A–C) The infiltration abundance of immune cells in low- and high-risk groups from the TCGA-LIHC, GSE14520 and LIRI-JP cohorts, respectively. (D) Relationships between overall survival and T cells gamma delta, T cells CD8 and macrophages M0 in the TCGA-LIHC cohort. (E) Relationships between overall survival and macrophages M1, B cells naive and macrophages M0 in the GSE14520 cohort. *p < 0.05; **p < 0.01; ***p < 0.001; ****p < 0.0001; ns, not significant.

We identified the abundance of 22 infiltrating immune cells in each HCC patient between low- and high-risk groups according to the CIBERSORT algorithm. In the TCGA-LIHC cohort, the infiltration abundance of macrophages M1, B cells naive, macrophages M2 and T cells gamma delta in the low-risk group were remarkably higher than those in the high-risk group (*p* < 0.01, Fig. 8A), whereas the infiltration abundance of B cells memory, T cells regulatory (Tregs), macrophages M0 and dendritic cells resting in the low-risk group were significantly lower than those in the high-risk group (*p* < 0.05, Fig. 8A). In the GSE14520 cohort, the abundance of B cells naive, Macrophages M1 as well as T cells gamma delta were significantly increased in the low-risk group compared to the high-risk group (*p* < 0.05, Fig. 8B); conversely, the abundance of macrophages M0, B cells memory and T cells regulatory (Tregs) were remarkably higher in the high-risk group compared to the low-risk group (*p* < 0.05, Fig. 8B). In the LIRI-JP cohort, the infiltration abundance of macrophages M1 was remarkably increased in the low-risk group compared with the high-risk group, while the infiltration abundance of neutrophils was significantly decreased (*p* < 0.01, Fig. 8C). Interestingly, we observed a significantly higher infiltration abundance of macrophages M1 in the low-risk group compared with the high-risk group in all three cohorts (*p* < 0.05).

Furthermore, we explored the prognostic value of 22 infiltrating immune cells, and in the TCGA-LIHC cohort, the infiltration abundance of T cells gamma delta, T cells CD8 and macrophages M0 were significantly correlated with OS (*p* < 0.05, Fig. 8D). Higher infiltration abundance of macrophages M0 was associated with worse overall survival, whereas higher infiltration abundance of T cells CD8 and T cells gamma delta were related to better OS. In the GSE14520 cohort, the infiltration abundance of macrophages M1, macrophages M0 and B cells naive were significantly associated with OS in HCC patients (*p* < 0.05, Fig. 8E). The higher infiltration abundance of macrophage M0 showed poorer overall survival, whereas the higher infiltration abundance of macrophages M1 and B cells naive showed better overall survival.

**Figure 9:**
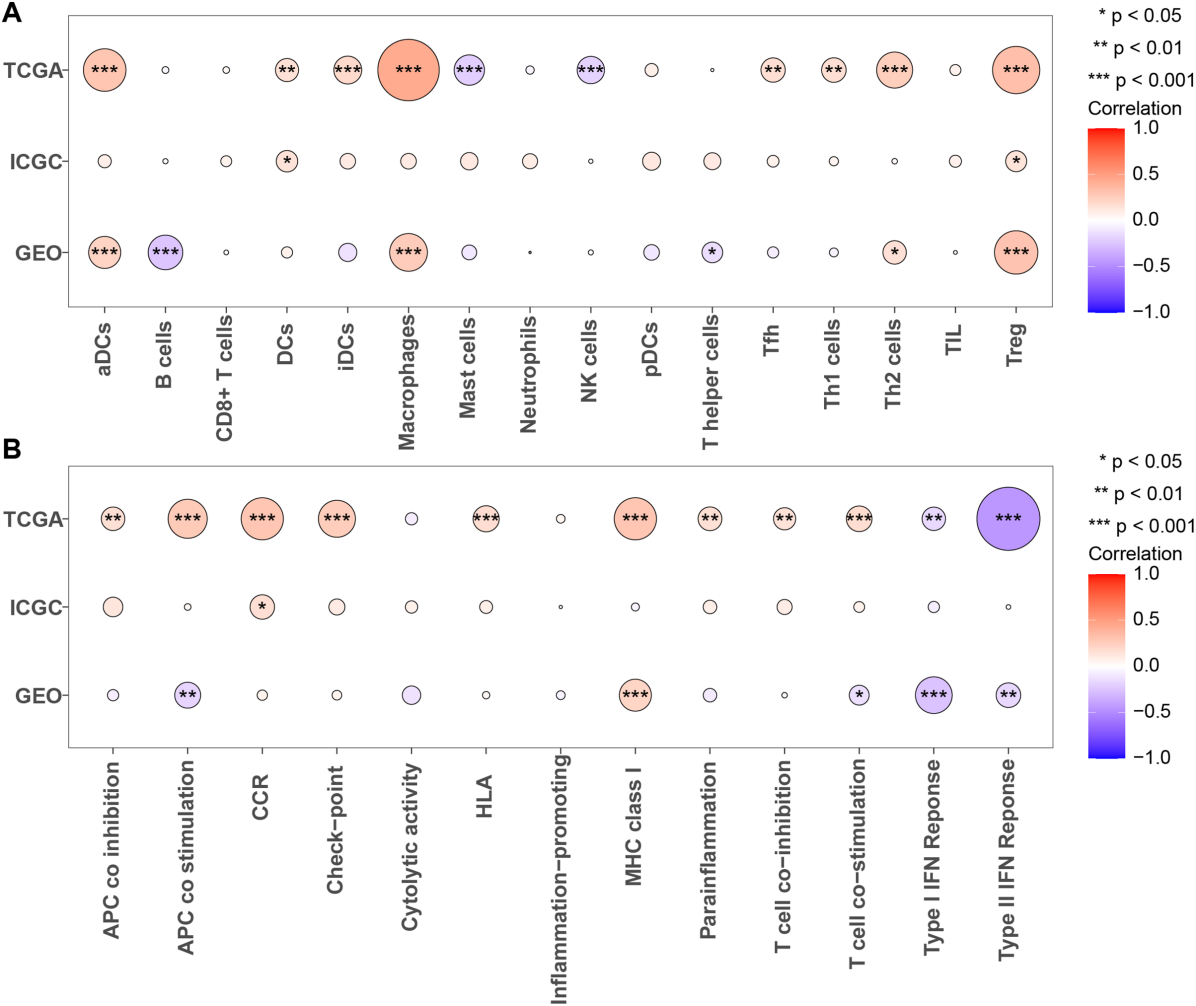
Evaluation of immune status in low- and high-risk groups. (A, B) Associations between PRGS and 16 immune cells and 13 immune functions were analyzed in the TCGA-LIHC, GSE14520 and LIRI-JP cohorts, respectively. *p < 0.05; **p < 0.01; ***p < 0.001.

Next, we calculated the enrichment scores for 13 immune functions and 16 immune cells in three cohorts using ssGSEA to further evaluate the association between PRGS and the scores of 13 immune functions and 16 immune cells (Figs. 9A and 9B). The results showed that Tregs were significantly positively correlated with PRGS in three cohorts (*p* < 0.05). The lower PRGS was closely related to B cells and NK cells. In immune functions, the higher PRGS was strongly associated with chemokine receptor (CCR) and immune checkpoints. These results demonstrated that PRGS was associated with many immune cells and immune functions, implying that PRGS could potentially reflect the tumor immune microenvironment.

### Immune checkpoints and drug susceptibility analysis

**Figure 10:**
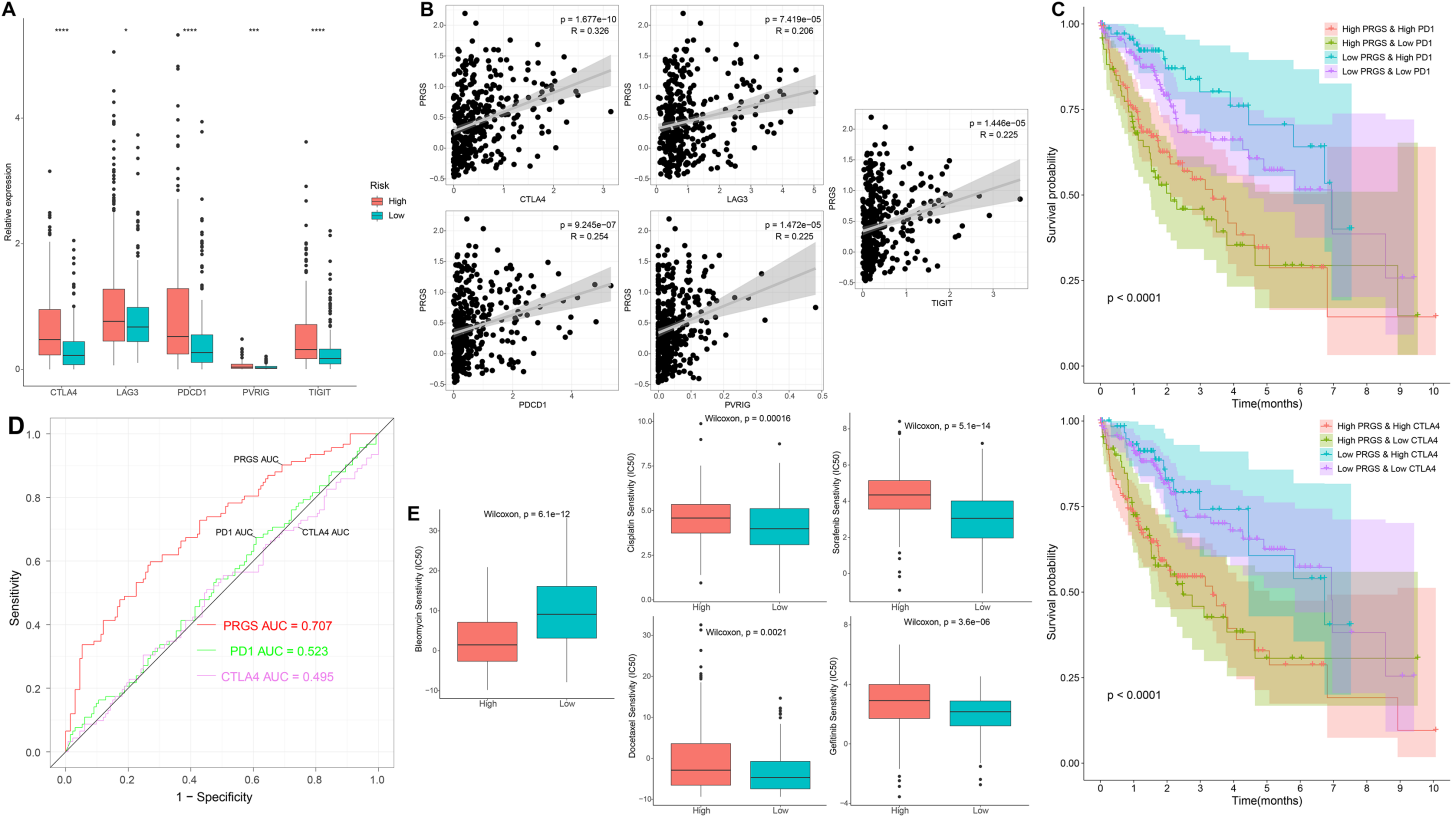
Immune checkpoints and drug susceptibility analysis. (A) Differential expression of immune checkpoints between low- and high-risk groups of the TCGA-LIHC cohort. (B) Correlations between PRGS and immune checkpoints. (C) Kaplan–Meier curves of OS in four groups based on PRGS and PD1/CTLA4. (D) ROC curves for the predictive power of PRGS, PD1 and CTLA4. (E) Relationships between PRGS and sensitivity to targeted therapy and chemotherapy drugs. *p < 0.05; ***p < 0.001; ****p < 0.0001.

To further examine the association of PRGS with immune checkpoints, we evaluated differences in immune checkpoints in low- and high-risk groups. In the training cohorts, immune checkpoints were expressed at higher levels in the high-risk group (*p* < 0.05, Fig. 10A). Overexpressed immune checkpoints promote tumor immune escape, which also explains that elevated immune checkpoints expression is associated with poorer prognosis in HCC patients (Xu et al., 2018; Zheng et al., 2021). In addition, the correlation analysis between PRGS and immune checkpoints showed that PRGS was significantly positively correlated with PD1, CTLA4, LAG3, PVRIG and TIGIT (*p* < 0.01, Fig. 10B).

In addition, we utilized IPS scores to determine the patient response to ICIs treatment. Four subtypes of IPS scores were performed to forecast response to anti-PD1 and anti-CTLA4 therapy in HCC patients. We found higher IPS scores in the low-risk group, indicating that patients in the low-risk group may respond better to immunotherapy (Fig. S9). Due to the complexity of ICIs in the tumor immune microenvironment, we further examined whether ICIs have an impact on the prognostic prediction of PRGS. As illustrated in Fig. 10C, the low-risk group with high PD1 exhibited better OS compared with the high-risk group and high PD1, and the OS of the low-risk group with low PD1 was longer than that of the high-risk group and low PD1 (*p* < 0.0001). Similarly, CTLA4 exhibited a survival prediction consistent with PD1 (*p* < 0.0001, Fig. 10C). Then, we utilized ROC curves to compare the prognostic predictive power of PRGS and ICIs, and the results showed that PRGS was superior to PD1 and CTLA4 (Fig. 10D).

We next selected targeted therapy and chemotherapy drugs currently utilized to treat HCC to evaluate the sensitivity of low- and high-risk groups to these drugs. Interestingly, the results showed that the IC50 values of bleomycin were absolutely decreased in the high-risk group, whereas the IC50 values of targeted therapy and chemotherapy drugs such as gefitinib, sorafenib, docetaxel and cisplatin were remarkably lower in the low-risk group (*p* < 0.01, Fig. 10E). Taken together, the above results suggested that PRGS was associated with drug sensitivity.

### The role of PRGS in immunotherapy

**Figure 11:**
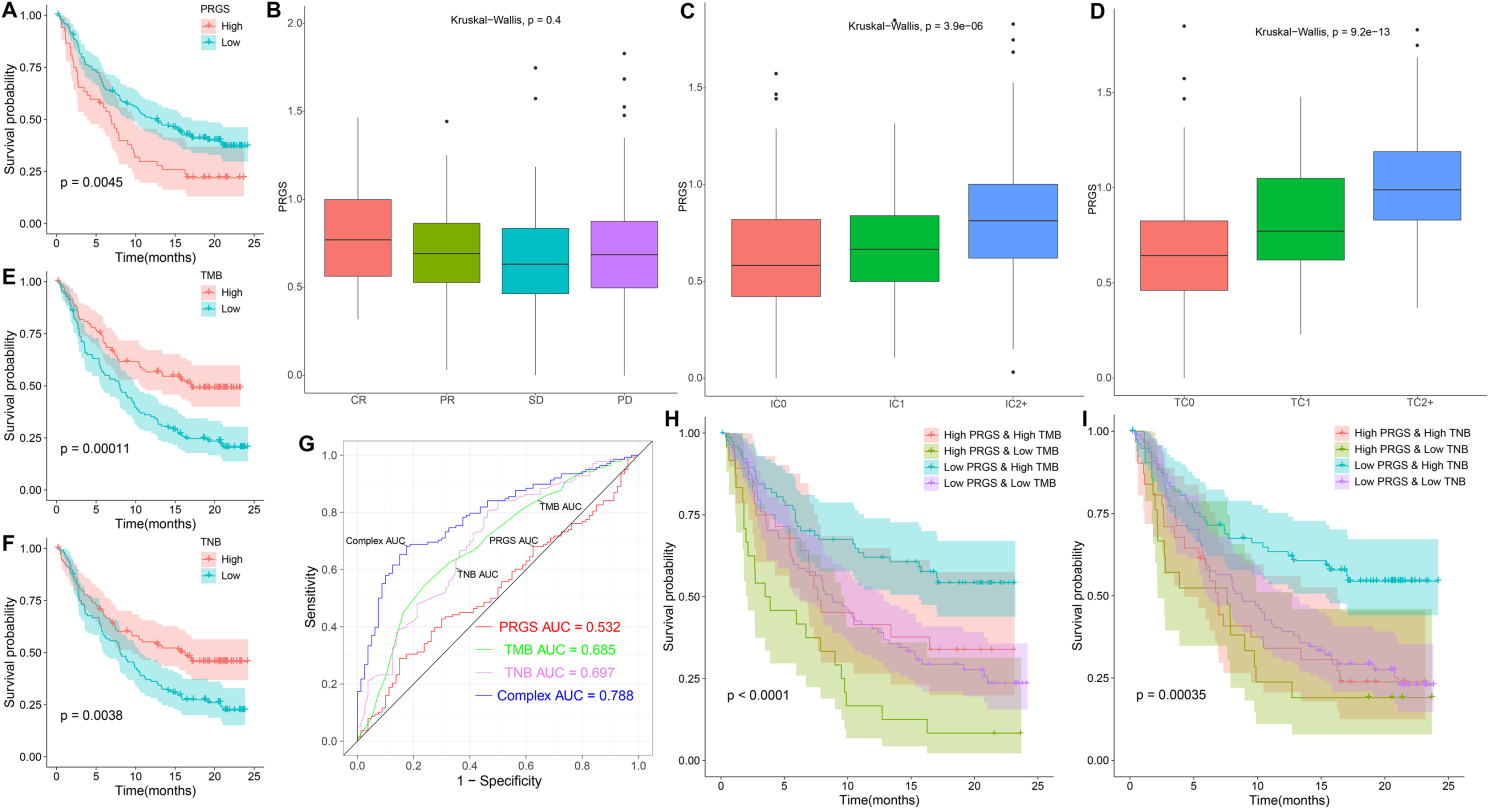
The role of PRGS in immunotherapy. (A) Kaplan–Meier curves of overall survival in the IMvigor210 cohort grouped into low- and high-risk groups. (B–D) PRGS were grouped by immunotherapy response, immune cell and tumor cell subgroups. (E, F) Kaplan–Meier curves of OS in the IMvigor210 cohort divided into low- and high-TMB/TNB groups. (G) ROC curves of PRGS, TMB, TNB and their combination (PRGS, TMB and TNB). (H–I) Kaplan–Meier curves of OS in four groups based on PRGS and TMB/TNB.

Given the relevance of PRGS to the tumor immune microenvironment, we further validated the effect of PRGS on immunotherapy efficacy in an immunotherapy cohort (IMvigor210). In order to make PRGS more convenient for the determination of low- and high-risk in patients receiving immunotherapy, we need a cut-off value to assist. Here, the cut-off value was 0.915, which is the optimum cut-off value obtained using the “survminer” R package. Patients in the IMvigor210 cohort were grouped into low-risk (PRGS < 0.915) and high-risk (PRGS > 0.915) groups based on the cut-off value, and we found that patients in the low-risk group exhibited better OS compared to those in the high-risk group (*p* = 0.0045, Fig. 11A). Not surprisingly, applying the cut-off value to the training and testing cohorts described above, the results also showed that the OS of patients in the low-risk group was superior to that in the high-risk group (*p* < 0.01, Fig. S10). Moreover, we also analyzed the relationship between PRGS and immunotherapy response, immune cell and tumor cell subgroups, and the results indicated that PRGS was not associated with immunotherapy response subgroups (Fig. 11B), but was associated with immune cell and tumor cell subgroups (*p* < 0.01, Figs. 11C and 11D). Interestingly, we found that PRGS was also positively correlated with immune checkpoints in the IMvigor210 cohort (*p* < 0.05, Fig. S11).

Growing evidence suggests that cancer patients with higher tumor mutation burden (TMB) might benefit more from immunotherapy because of their higher tumor neoantigen burden (TNB) (Hollern et al., 2019). In the IMvigor210 cohort, high TMB and TNB represented better overall survival (*p* < 0.01, Figs. 11E and 11F). ROC curve analysis showed that the AUC value (0.788) obtained by the combination of TMB, TNB and PRGS was higher than that of TMB (0.685), TNB (0.697) or PRGS (0.532) alone (Fig. 11G). Next, we further evaluated the impact of TMB and TNB on the prognostic prediction of PRGS. Figure 11H showed that the low-PRGS with high TMB exhibited better overall survival compared with high-PRGS and high TMB, and the overall survival of the low-PRGS with low TMB were superior to that of high-PRGS and low TMB (*p* < 0.0001). Similarly, TNB exhibited a survival prediction consistent with TMB (*p* = 0.00035, Fig. 11I), and PRGS combined with TMB or TNB predicted more significant results than either alone. These results suggested that considering PRGS together with TMB and TNB could improve the accuracy of assessing the efficacy of immunotherapy and determine patients who will respond better to immunotherapy.

## Discussion

Early diagnosis and prognosis prediction of HCC are of great importance for patient survival. Currently, the diagnosis and prognosis prediction of HCC are mostly according to pathological assessment and BCLC staging, but these methods are not sensitive enough (Giannini et al., 2018; Sherman, 2014). Therefore, the search for effective biomarkers to develop accurate and reliable prognostic model is urgent to improve the survival of HCC patients. Earlier studies have pointed out the integral role of pyroptosis in immune response and tumor therapy, including HCC (Tan et al., 2021; Wang et al., 2021). However, several previous studies only considered individual pyroptosis-related gene or tumor-infiltrating immune cell, without exploring the overall function of multiple PRGs in HCC and their relationship with the immune microenvironment (Pang et al., 2021; Shangguan et al., 2021). This study systematically compared the genetic and expression alterations of PRGs in HCC. We determined two distinct pyroptosis-related subtypes (cluster 1 and cluster 2) based on differentially expressed PRGs using a consensus clustering algorithm. Cluster 1 patients had more advanced clinical characteristics and poorer overall survival compared to cluster 2 patients. We extracted pyroptosis-related DEGs in cluster 1 and cluster 2 for GO and KEGG analysis. Next, to assess the prognosis of HCC patients, we constructed a prognostic prediction model PRGS by implementing univariate Cox and LASSO Cox analyses of pyroptosis-related DEGs, which exhibited excellent predictive power in both the training and testing cohorts. Considering that PRGS was constructed using CYP2C9, MYBL2, SPP1, CTSV and EPO, we then validated the differences in their expression levels in normal and HCC liver tissues. Using protein expression data from HPA, the expression levels of MYBL2, SPP1, CTSV and EPO were increased in HCC tissues compared with normal liver tissues, while the expression level of CYP2C9 was decreased. Moreover, the expression of these five genes was verified in human normal liver cells and liver cancer cells. Patients in low- and high-risk groups identified according to PRGS exhibited significantly different clinical characteristics, prognosis, immune microenvironment, immune checkpoints and drug susceptibility. Moreover, PRGS produced significant survival differences in patients with various subgroups of clinical characteristics, with the exception of the female subgroup. Later, by integrating PRGS and clinical characteristics, we constructed nomograms to further enhance the predictive power and facilitate the application of PRGS. Importantly, the CIBERSORT results revealed higher infiltration abundance of T cells gamma delta, macrophages M1 and B cells naive, but lower infiltration abundance of T cells regulatory (Tregs) and macrophages M0 in the low-risk group; ssGSEA analysis indicated that low-PRGS was characterized by higher enrichment scores for immune cells (NK cells and B cells) and immune functions (type I and II IFN responses); additionally, immune checkpoint expression levels in the high-risk group were higher than those in the low-risk group, indicating potential differences in the immune microenvironment between low- and high-risk groups. Overall, lower and higher PRGS showed immune activation and inhibition, respectively. Notably, the higher infiltration abundance of macrophages M0 showed poorer survival, whereas the higher infiltration abundance of T cells CD8, macrophages M1 and B cells naive were associated with better survival. Finally, our application of PRGS to an immunotherapy cohort was also able to predict overall survival of patients, and found that combining PRGS with TMB and TNB could enhance the assessment of immunotherapy efficacy.

Currently, pyroptosis has received more and more attention in tumor research (Li et al., 2021a; Wu et al., 2021a). Importantly, pyroptosis can release inflammatory factors to suppress tumorigenesis, and can also develop a microenvironment conducive to tumor cell proliferation and promote tumor development (Xia et al., 2019). Thus, pyroptosis is considered to have a critical role in tumor therapy and prognosis prediction. However, the role of pyroptosis in the prognosis of HCC patients is still not fully defined. We developed an independent prognostic prediction model for HCC using five prognostic genes of PRGS, including CYP2C9, MYBL2, SPP1, CTSV and EPO. CYP2C9, a member of the CYP450 family, has been found to be a potential prognostic biomarker for HCC (Wang et al., 2018). CYP2C9 is downregulated in HCC, and its inhibition is closely related to the progression of HCC (Hu et al., 2019; Yu et al., 2015). As a physiological regulator, MYBL2 is frequently found to be dysregulated in tumors, accelerating tumorigenesis and progression (Musa et al., 2017). MYBL2 is overexpressed in HCC and is an independent prognostic factor of unfavorable OS (Guan et al., 2018). Mechanistically, MYBL2 induces HCC progression by regulating the cell cycle and activating genes and pathways associated with tumorigenesis (Frau et al., 2011). SPP1 is a protein-coding gene that is upregulated in HCC with a close relation to the tumor process (Wang et al., 2019a). A study suggests that SPP1 is a predictor of adverse prognosis in HCC patients and that targeting SPP1 might be a potential approach to modulate the tumor microenvironment and enhance response to immunotherapy in HCC (Liu et al., 2022). CTSV, a lysosomal cysteine protease, is expressed at increased levels in HCC and is an independent prognostic risk factor for HCC survival, and elevated CTSV expression is strongly correlated with an adverse prognosis in HCC (Jing et al., 2018). EPO is a pleiotropic cytokine that regulates multiple cellular activities and attenuates pyroptosis (Yang et al., 2021). EPO is increased in HCC and its production is inversely correlated with the overall prognosis of HCC (Ke et al., 2017). To sum up, consistent with our results, these studies demonstrate the impact of these five genes on the prognosis of HCC and might be targets for HCC treatment.

Poor prognosis of HCC after traditional chemotherapy due to heterogeneity of infiltrating immune cells and immune checkpoints (Song et al., 2021). HCC has made advances in immunotherapy in recent years, but the prognosis of patients is still heterogeneous, which emphasizes the crucial role of the TME in the progression and prognosis of HCC (Llovet et al., 2021a). The main immune cells in the immune microenvironment include macrophages, lymphocytes and granulocytes. These immune cells are involved in a series of immune activities and responses, including tumor suppression and promotion (Arneth, 2019). Moreover, the immune microenvironment is also involved in tumor growth, metastasis, and therapeutic resistance (Mehraj et al., 2021). In this study, immune activation was associated with lower PRGS, whereas immune inhibition was associated with higher PRGS. We found that the infiltration abundance of many immune cells differed significantly between low- and high-risk groups. Growing evidence suggests that T cell-mediated immune responses play a critical role in the tumor immune microenvironment of HCC (Bian et al., 2020). The density of tumor-infiltrating T cells correlates with superior HCC patient survival, with higher density indicating a good prognosis (Garnelo et al., 2017). Notably, T cells gamma delta can infiltrate HCC, recognize and kill tumor cells, showing potent tumor-killing capacity (Jiang et al., 2019). In this study, the low-risk group had a better prognosis, showing a higher infiltration abundance of T cells gamma delta, suggesting that it plays a positive role in HCC prognosis. Moreover, higher infiltration abundance of T cells gamma delta and T cells CD8 in the immune microenvironment was correlated with a favorable prognosis of HCC. Tregs infiltration inhibits T cell activation as well as anti-tumor immune response, which are related to adverse prognosis of HCC (Tu et al., 2016). This is consistent with our finding that the infiltration abundance of Tregs was higher in the high-risk group compared to the low-risk group. B cells are the main effector cells of the adaptive immune response, and many studies have suggested that B cells play a vital role in tumor immunity (Tokunaga et al., 2019; Wang et al., 2019b). B cells in tumors are considered to be predictors of patient prognosis, and studies have found that B cells have dual role in tumors, including pro-tumor and anti-tumor effects (Bruno, 2020; Llovet et al., 2021a; Shalapour et al., 2015). This study exhibited that the low-risk group had a higher infiltration abundance of B cells naive and a lower infiltration abundance of B cells memory compared with the high-risk group, suggesting a potential dual role for B cells. Among macrophage subsets, macrophages M1 that produce proinflammatory cytokines have anti-tumor effects (Zhou et al., 2020). The study found that macrophages M1 can inhibit the growth, invasion and metastasis of HCC and advance apoptosis of HCC cells (Bai et al., 2020). Furthermore, higher macrophages M1 is related to better overall survival (Shu et al., 2016). Consistent with published studies, we noted a higher infiltration abundance of macrophages M1 in the low-risk group and represented a better prognosis.

At present, targeted therapy is an important clinical treatment option for HCC. Thus, there is an urgent need to determine patients who might benefit from targeted therapy for HCC. We assessed the IC50 of two targeted therapy drugs commonly utilized in the first-line treatment of HCC. Interestingly, the results showed a higher sensitivity to sorafenib and gefitinib in the low-risk group than in the high-risk group. Meanwhile, immunotherapy offers a new direction for the treatment of HCC. For immunotherapy exploration, anti-CTLA4, anti-PD1 and anti-PD-L1 therapy have become the focus of current attention. However, a minority of patients respond to such treatments, and several studies suggest that PD1 and PD-L1 are not reliable biomarkers for predicting ICIs treatment (Fuchs et al., 2018; Shen & Zhao, 2018). Therefore, it is necessary to establish a reliable tool to forecast immunotherapy efficacy in HCC patients. According to the findings in our research, we identified that PRGS is a powerful immune classifier that can be applied to predict the prognosis of immunotherapy.

Our study linked pyroptosis with the prognosis of HCC patients to construct a prognostic prediction model PRGS, and outperformed some other published pyroptosis-related signatures (**Table S4**) (Fu & Song, 2021; Jin et al., 2022; Liu et al., 2021; Wu et al., 2021b), which also indicated the superiority of the model PRGS. Moreover, compared with other studies, our study has the following advantages: first, we compared the predictive power of different models and the results showed that our model PRGS was superior to other models; second, most studies only used one validation cohort, while our study used multiple validation cohorts and the model was more robust; third, we fully evaluated the tumor immune microenvironment; fourth, we also used an immunotherapy cohort to evaluate the PRGS model for the prediction of immunotherapy prognosis; fifth, we also conducted *in vitro* experiments to validate. Taken together, the current study comprehensively assessed the potential link between pyroptosis, prognosis and immune microenvironment in HCC patients, providing novel ideas for improving HCC prognosis and treatment. Nevertheless, our current study had some limitations. Frist, the data sources for this study are mainly public databases. Extensive clinical data is required to validate the predictive power of this model, and we have begun collecting clinical validation samples. Second, our prognostic prediction model is related to immune cells, but the mechanism is not yet clear and requires extra *in vitro* and *in vivo* experimental studies to further explore, which is also an aspect we need to investigate in the future.

## Conclusions

Our comprehensive analysis of PRGS revealed its relationship with the immune microenvironment, clinical characteristics and prognosis in HCC. We also identified the efficacy prediction of PRGS in immunotherapy and targeted therapy. These results emphasize the critical clinical significance of PRGS in HCC survival prediction and individualized treatment guidance.

## Supplemental Information

10.7717/peerj.14691/supp-1 Supplemental Information 1 Supplementary Figures and Tables.

10.7717/peerj.14691/supp-2 Supplemental Information 2 Raw data for cell line experiments.

## Additional Information and Declarations

### Data Availability

The following information was supplied regarding data availability: The raw data is available at figshare: Wang, Sheng (2022): Raw_data.zip. figshare. Dataset. https://doi.org/10.6084/m9.figshare.21732488.v1.
