# Supplementary material for: Comprehensive genomic signature of pyroptosis-related genes and relevant characterization in hepatocellular carcinoma
Source: PeerJ. 2023 Jan 12;11:e14691. doi: 10.7717/peerj.14691 (PMC9840857; doi:10.7717/peerj.14691)
Supplement: Supplemental Information 1 [file peerj-11-14691-s001.zip › Supplementary materials/Supplementary Figure Legends.docx]

**SUPPLEMENTLY FIGURE LEGENDS**

**Figure S1** The workflow chart of our study.

**Figure S2** Consensus Clustering analysis. Cumulative distribution function (CDF) based on differentially expressed PRGs indicated that the optimal number of pyroptosis-related subtypes was 2.

**Figure S3** Results of Silhouette analysis. Silhouette analysis was used to measure how close each point was to one cluster than to the other to measure distinct types according to differentially expressed PRGs.

**Figure S4** LASSO Cox regression and PCA analysis. **(A)** In LASSO Cox regression, LASSO coefficients for prognostic genes were obtained by 10-fold cross-validation using the minimum criterion. **(B)** PCA plot showing that low- and high-risk groups were clustered separately.

**Figure S5** ROC curves for the predictive power of PRGS and other published pyroptosis-related signatures in the testing cohorts (left: LIRI-JP; right: GSE14520).

**Figure S6** The expression levels of 5 prognostic genes in Huh7 and HepG2 cell lines.

**Figure S7** Predictive power of PRGS in different subgroups of clinical characteristics in the TCGA cohort. The Kaplan-Meier curve of overall survival in low- and high-risk groups based on age, gender, stage, grade, T stage, and fibrosis.

**Figure S8** Construction and validation of the nomogram to predict the overall survival in the testing cohort. **(A-B)** Nomograms containing PRGS to predict the overall survival in the LIRI-JP and GSE14520 cohorts. **(C-D)** Calibration plot of the nomogram on consistency between predicted and observed 1-, 3-, and 4-year survival in the LIRI-JP and GSE14520 cohorts. **(E-F)** Time-dependent ROC curves for predicting 1-, 3-, and 4-year survival in the LIRI-JP and GSE14520 cohorts using the nomogram. **(G-I)** The AUC values of nomogram for PRGS and other published pyroptosis-related signatures in the training and testing cohorts (TCGA-LIHC, LIRI-JP and GSE14520).

**Figure S9** Responses to immune checkpoint inhibitors. Violin plots illustrate anti-PD1 and anti-CTLA4 treatment responses between low- and high-risk groups.

**Figure S10** Applying the cut-off value to the training and testing cohorts. Kaplan-Meier curves of overall survival in the training and testing cohorts divided into low- and high-risk groups.

**Figure S11** Correlations between PRGS and immune checkpoints in the IMvigor210 cohort. PRGS was positively correlated with immune checkpoints.
