# Supplementary material for: Comprehensive genomic signature of pyroptosis-related genes and relevant characterization in hepatocellular carcinoma
Source: PeerJ. 2023 Jan 12;11:e14691. doi: 10.7717/peerj.14691 (PMC9840857; doi:10.7717/peerj.14691)
Supplement: Supplemental Information 1 [file peerj-11-14691-s001.zip › Supplementary materials/Supplementary Table S1.docx]

| Table S1: Summary of 48 pyroptosis-related genes | |
| --- | --- |
| **Gene** | **Type** |
| AIM2 | pyroptosis |
| CASP1 | pyroptosis |
| CASP3 | pyroptosis |
| CASP4 | pyroptosis |
| CASP5 | pyroptosis |
| CASP6 | pyroptosis |
| CASP8 | pyroptosis |
| CASP9 | pyroptosis |
| GPX4 | pyroptosis |
| GSDMA | pyroptosis |
| GSDMB | pyroptosis |
| GSDMC | pyroptosis |
| GSDMD | pyroptosis |
| GSDME | pyroptosis |
| IL18 | pyroptosis |
| IL1B | pyroptosis |
| IL6 | pyroptosis |
| NLRC4 | pyroptosis |
| NLRP1 | pyroptosis |
| NLRP2 | pyroptosis |
| NLRP3 | pyroptosis |
| NLRP6 | pyroptosis |
| NOD1 | pyroptosis |
| NOD2 | pyroptosis |
| PJVK | pyroptosis |
| PLCG1 | pyroptosis |
| PRKACA | pyroptosis |
| PYCARD | pyroptosis |
| SCAF11 | pyroptosis |
| TIRAP | pyroptosis |
| TNF | pyroptosis |
| GZMA | pyroptosis |
| GZMB | pyroptosis |
| BAK1 | pyroptosis |
| BAX | pyroptosis |
| CHMP2A | pyroptosis |
| CHMP2B | pyroptosis |
| CHMP3 | pyroptosis |
| CHMP4B | pyroptosis |
| CHMP4C | pyroptosis |
| CHMP6 | pyroptosis |
| CHMP7 | pyroptosis |
| CYCS | pyroptosis |
| HMGB1 | pyroptosis |
| IRF1 | pyroptosis |
| IRF2 | pyroptosis |
| TP53 | pyroptosis |
| TP63 | pyroptosis |
