# Supplementary material for: Comprehensive genomic signature of pyroptosis-related genes and relevant characterization in hepatocellular carcinoma
Source: PeerJ. 2023 Jan 12;11:e14691. doi: 10.7717/peerj.14691 (PMC9840857; doi:10.7717/peerj.14691)
Supplement: Supplemental Information 1 [file peerj-11-14691-s001.zip › Supplementary materials/Supplementary Table S2.docx]

| Table S2: Oligonucleotide primer sequences used in the RT-qPCR | | |
| --- | --- | --- |
| **Gene** | **Forward (5’ to 3’)** | **Reverse (5’ to 3’)** |
| CYP2C9 | CAGAGACGACAAGCACAACCCT | ATGTGGCTCCTGTCTTGCATGC |
| SPP1 | TCCTAGCCCCACAGACCCTT | CTGTGGAATTCACGGCTGAC |
| MYBL2 | CTTGAGCGAGTCCAAAGACTG | AGTTGGTCAGAAGACTTCCCT |
| EPO | GCATGTGGATAAAGCCGTCAGTG | GAGTTTGCGGAAAGTGTCAGCAG |
| CTSV | CGTGACGCCAGTGAAGAATCA | CGCTCAGTGAGACAAGTTTCC |
| β-actin | CACCATTGGCAATGAGCGGTTC | AGGTCTTTGCGGATGTCCACGT |
