# Supplementary material for: Comprehensive genomic signature of pyroptosis-related genes and relevant characterization in hepatocellular carcinoma
Source: PeerJ. 2023 Jan 12;11:e14691. doi: 10.7717/peerj.14691 (PMC9840857; doi:10.7717/peerj.14691)
Supplement: Supplemental Information 1 [file peerj-11-14691-s001.zip › Supplementary materials/Supplementary Table S4.docx]

| Table S4: The AUC values of PRGS and other published pyroptosis-related signatures | |
| --- | --- |
| **Training cohort (TCGA-LIHC)** |  |
| **Pyroptosis-related signatures** | **AUC values** |
| PRGS | 0.707 |
| PRGS1, PMID: 34820372 | 0.543 |
| PRGS2, PMID: 34820376 | 0.615 |
| PRGS3, PMID: 34869364 | 0.671 |
| PRGS4, PMID: 35659304 | 0.652 |
|  |  |
| **Testing cohort (LIRI-JP)** |  |
| **Pyroptosis-related signatures** | **AUC values** |
| PRGS | 0.701 |
| PRGS1, PMID: 34820372 | 0.487 |
| PRGS2, PMID: 34820376 | 0.504 |
| PRGS3, PMID: 34869364 | 0.515 |
| PRGS4, PMID: 35659304 | 0.559 |
|  |  |
| **Testing cohort (GSE14520)** |  |
| **Pyroptosis-related signatures** | **AUC values** |
| PRGS | 0.634 |
| PRGS1, PMID: 34820372 | 0.604 |
| PRGS2, PMID: 34820376 | 0.523 |
| PRGS3, PMID: 34869364 | 0.582 |
| PRGS4, PMID: 35659304 | 0.536 |
