# Supplementary material for: Comprehensive genomic signature of pyroptosis-related genes and relevant characterization in hepatocellular carcinoma
Source: PeerJ. 2023 Jan 12;11:e14691. doi: 10.7717/peerj.14691 (PMC9840857; doi:10.7717/peerj.14691)
Supplement: Supplemental Information 1 [file peerj-11-14691-s001.zip › Supplementary materials/Supplementary Table S5.docx]

| Table S5: Predictive power of nomograms of PRGS and other published pyroptosis-related signatures | | |
| --- | --- | --- |
| **Training cohort (TCGA-LIHC)** |  |  |
| **Pyroptosis-related signatures** | **AUC values** | **C-index** |
| PRGS | 0.729 | 0.715 |
| PRG1, PMID: 34820372 | 0.613 | 0.590 |
| PRG2, PMID: 34820376 | 0.678 | 0.662 |
| PRG3, PMID: 35659304 | 0.713 | 0.678 |
|  |  |  |
| **Testing cohort (LIRI-JP)** |  |  |
| **Pyroptosis-related signatures** | **AUC values** | **C-index** |
| PRGS | 0.750 | 0.769 |
| PRG1, PMID: 34820372 | 0.638 | 0.669 |
| PRG2, PMID: 34820376 | 0.701 | 0.728 |
| PRG3, PMID: 35659304 | 0.671 | 0.715 |
|  |  |  |
| **Testing cohort (GSE14520)** |  |  |
| **Pyroptosis-related signatures** | **AUC values** | **C-index** |
| PRGS | 0.705 | 0.702 |
| PRG1, PMID: 34820372 | 0.690 | 0.687 |
| PRG2, PMID: 34820376 | 0.656 | 0.647 |
| PRG3, PMID: 35659304 | 0.634 | 0.625 |
