# Supplementary figures and images for: Comprehensive genomic signature of pyroptosis-related genes and relevant characterization in hepatocellular carcinoma
Source: PeerJ. 2023 Jan 12;11:e14691. doi: 10.7717/peerj.14691 (PMC9840857; doi:10.7717/peerj.14691)

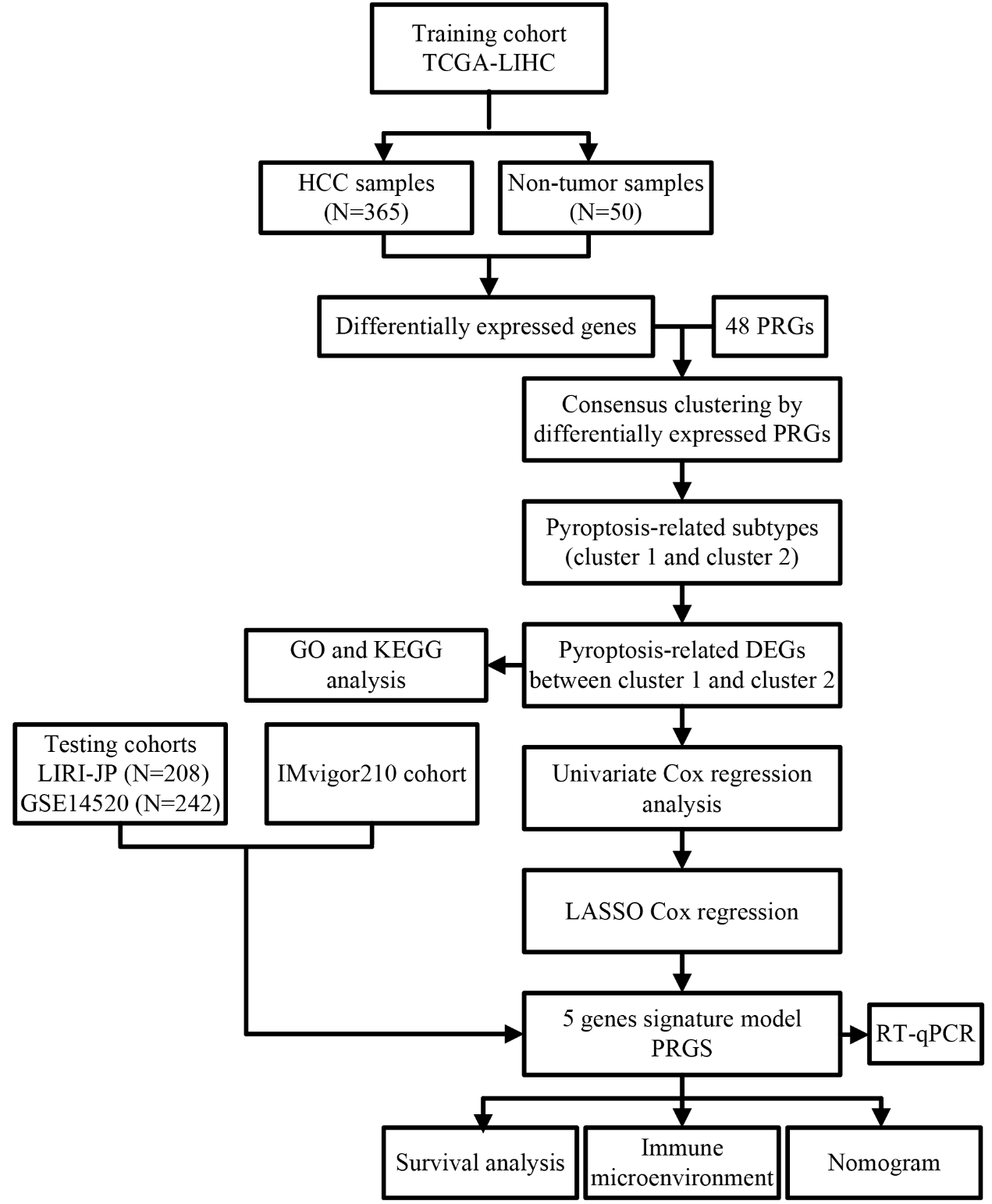

Supplement: Supplemental Information 1 [file peerj-11-14691-s001.zip › Supplementary materials/Figure S1.png]

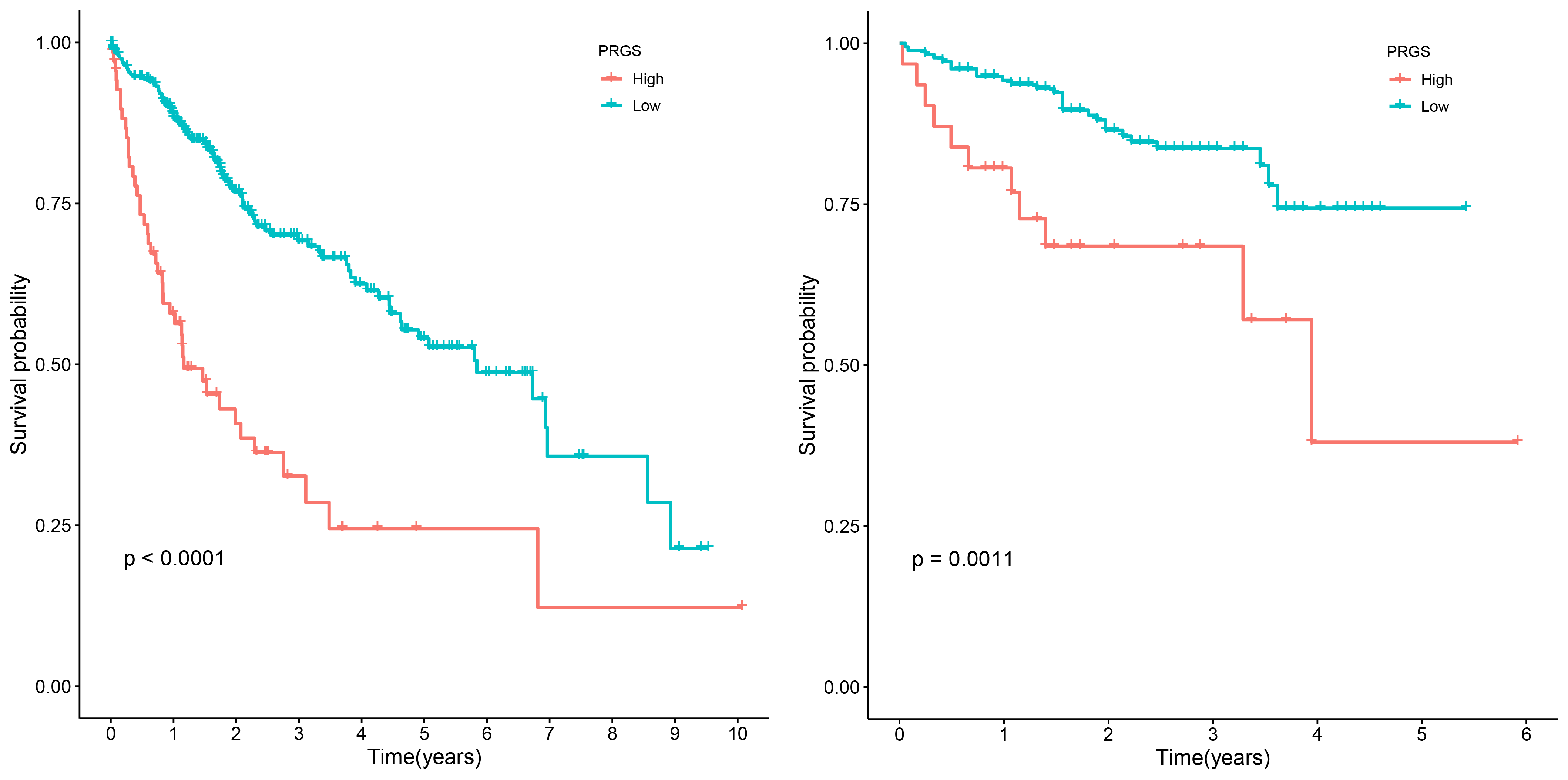

Supplement: Supplemental Information 1 [file peerj-11-14691-s001.zip › Supplementary materials/Figure S10.png]

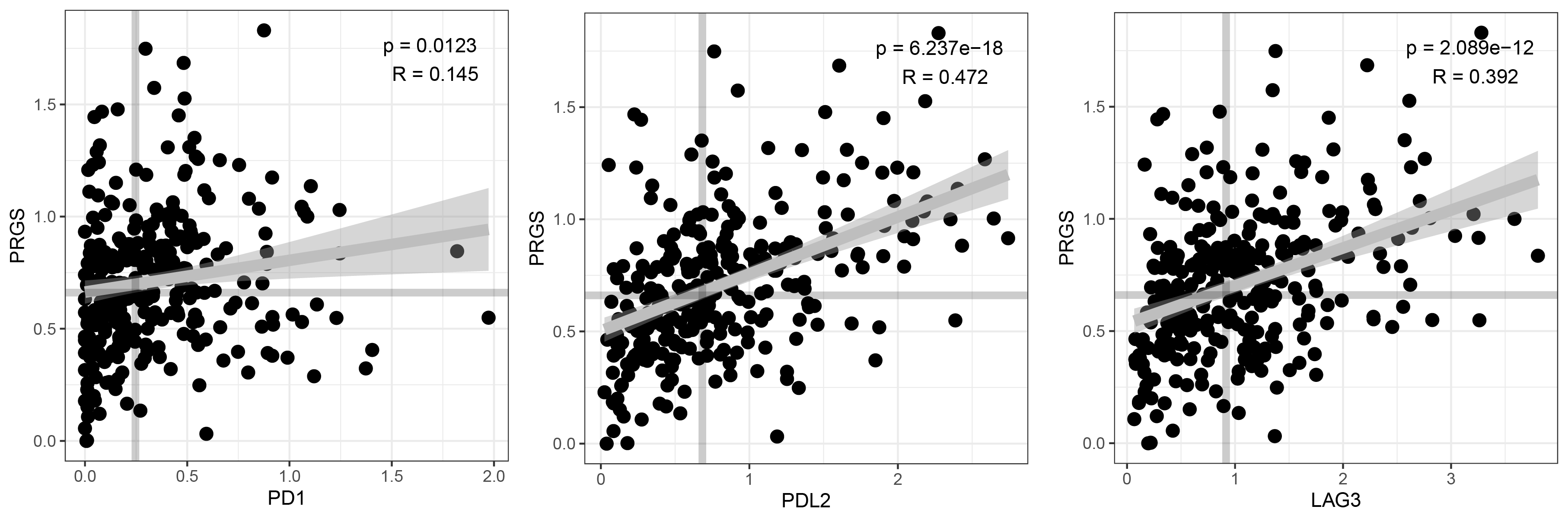

Supplement: Supplemental Information 1 [file peerj-11-14691-s001.zip › Supplementary materials/Figure S11.png]

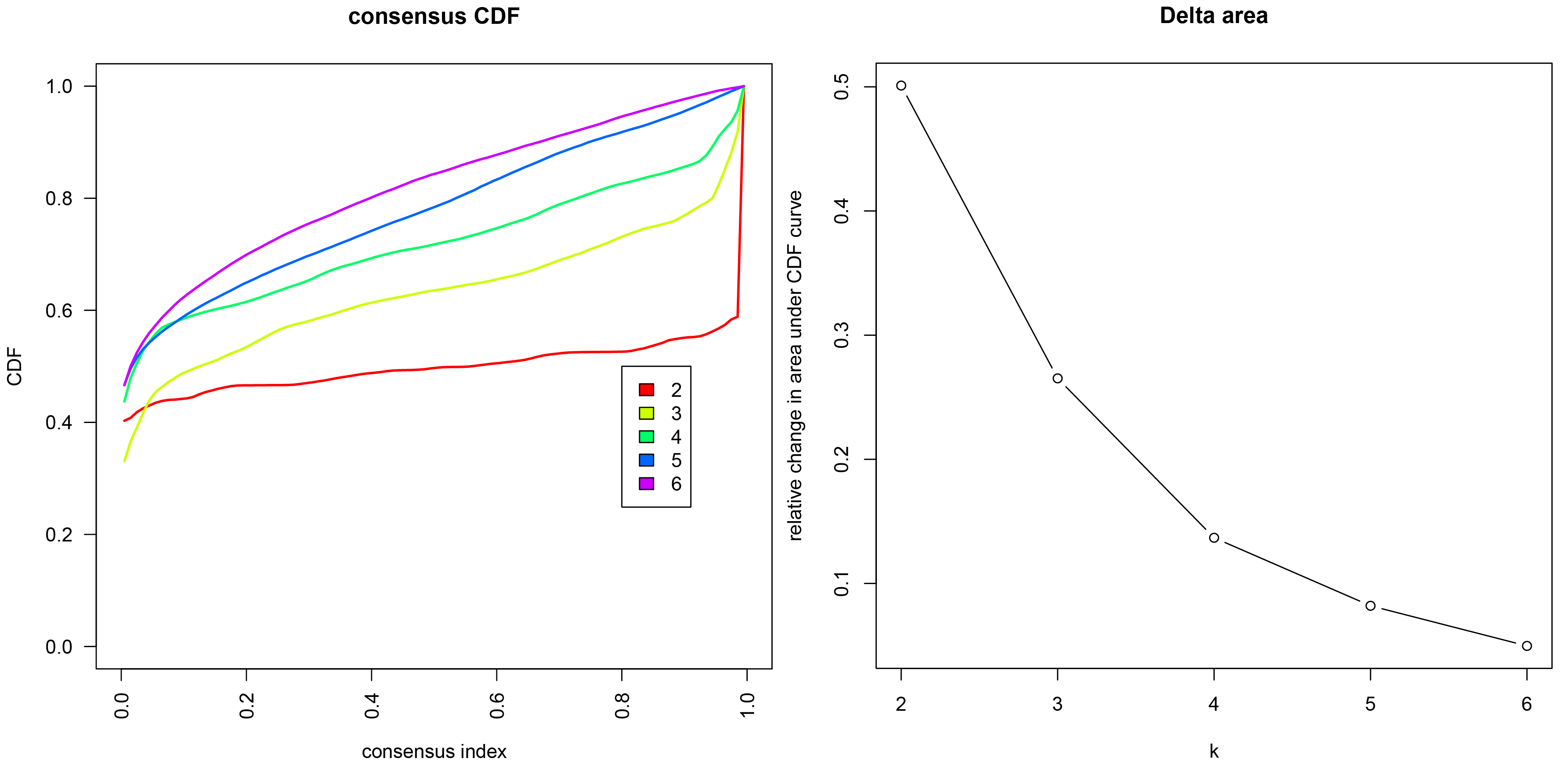

Supplement: Supplemental Information 1 [file peerj-11-14691-s001.zip › Supplementary materials/Figure S2.png]

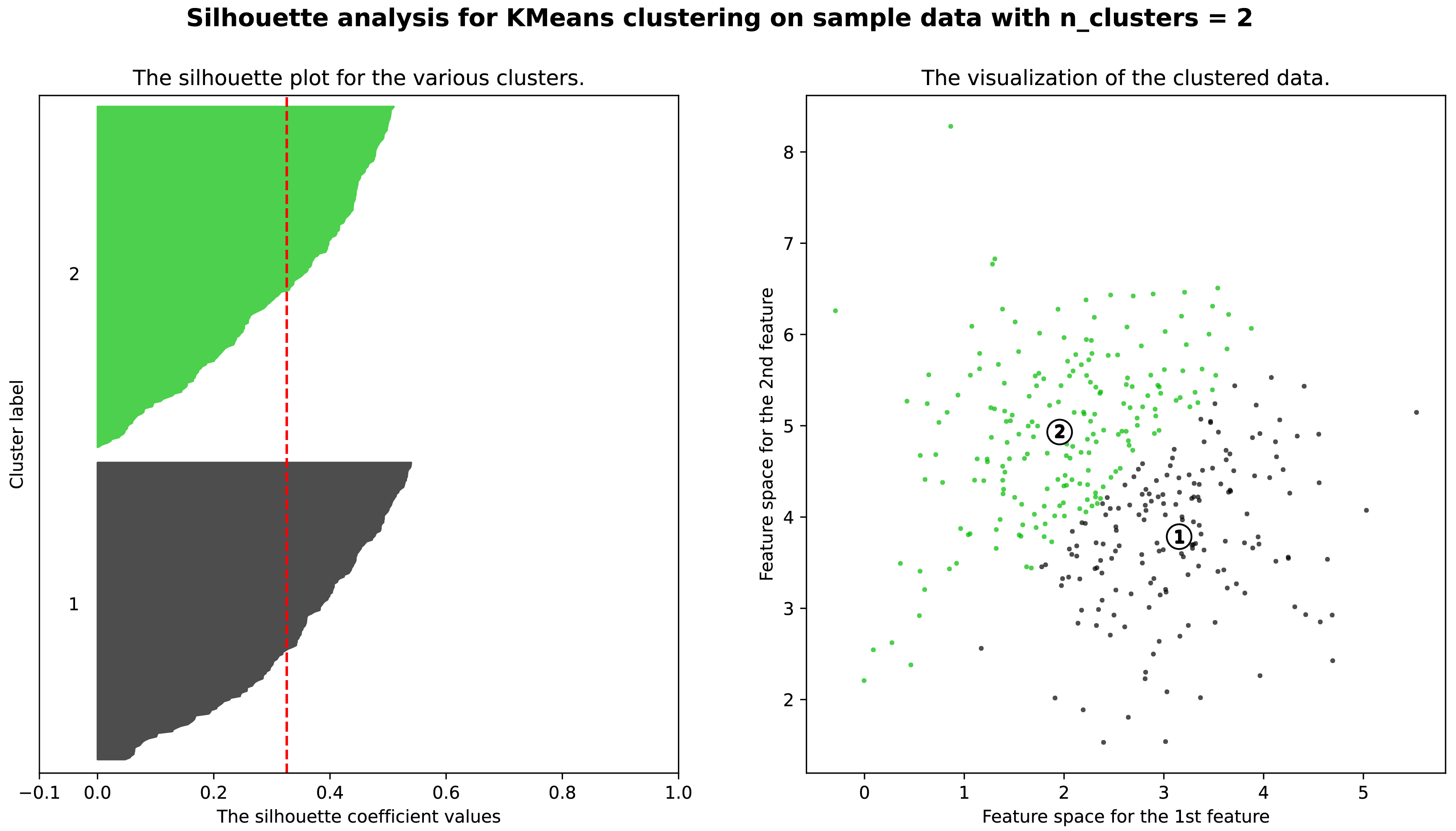

Supplement: Supplemental Information 1 [file peerj-11-14691-s001.zip › Supplementary materials/Figure S3.png]

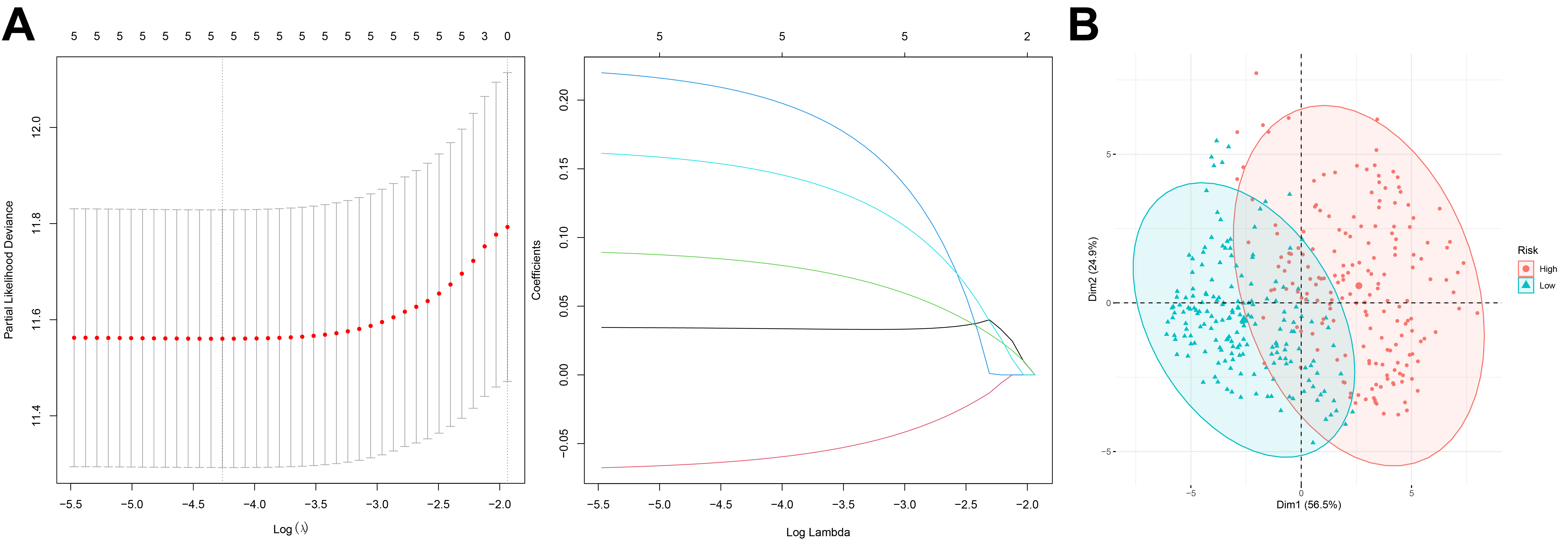

Supplement: Supplemental Information 1 [file peerj-11-14691-s001.zip › Supplementary materials/Figure S4.png]

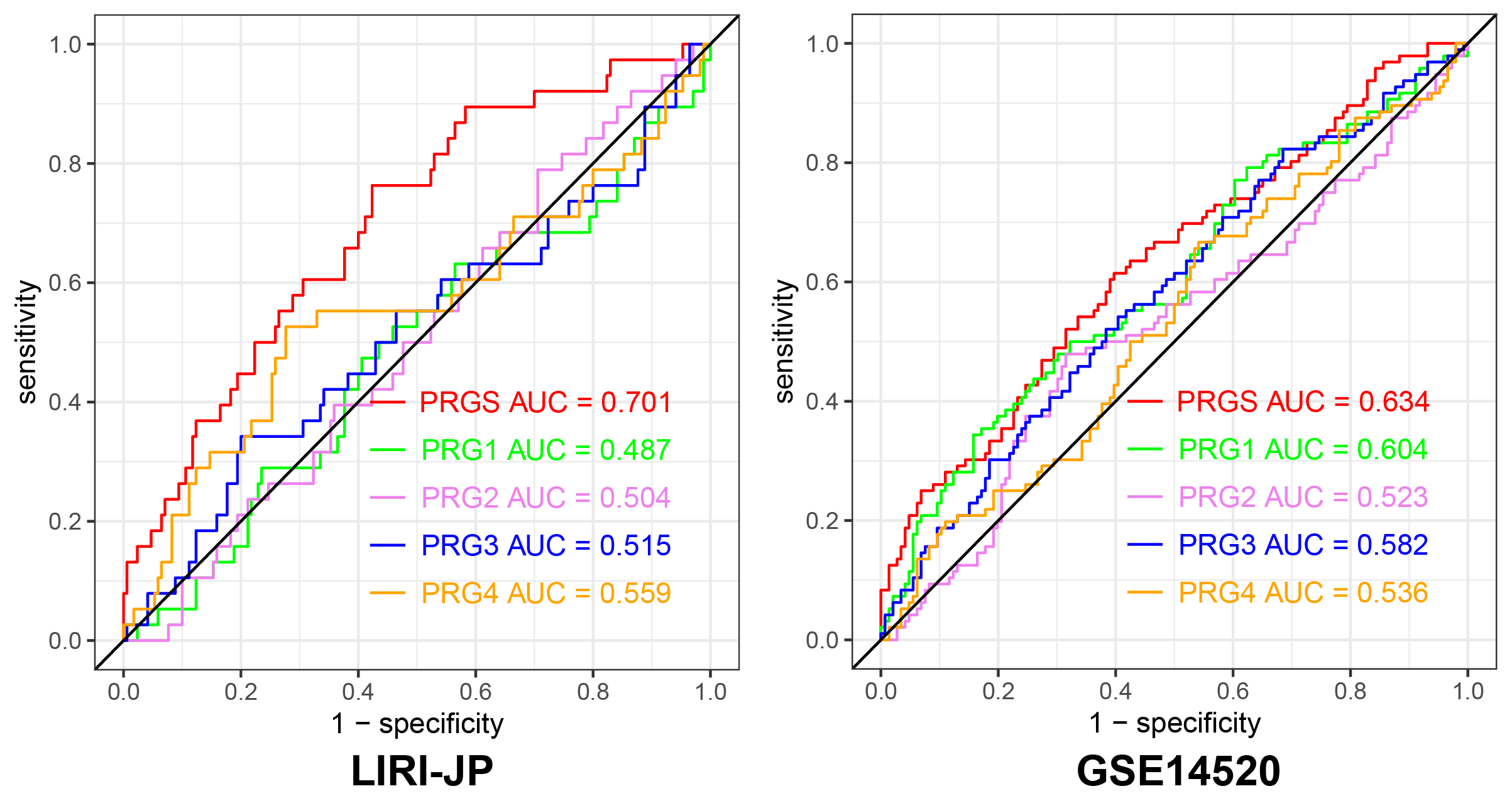

Supplement: Supplemental Information 1 [file peerj-11-14691-s001.zip › Supplementary materials/Figure S5.png]

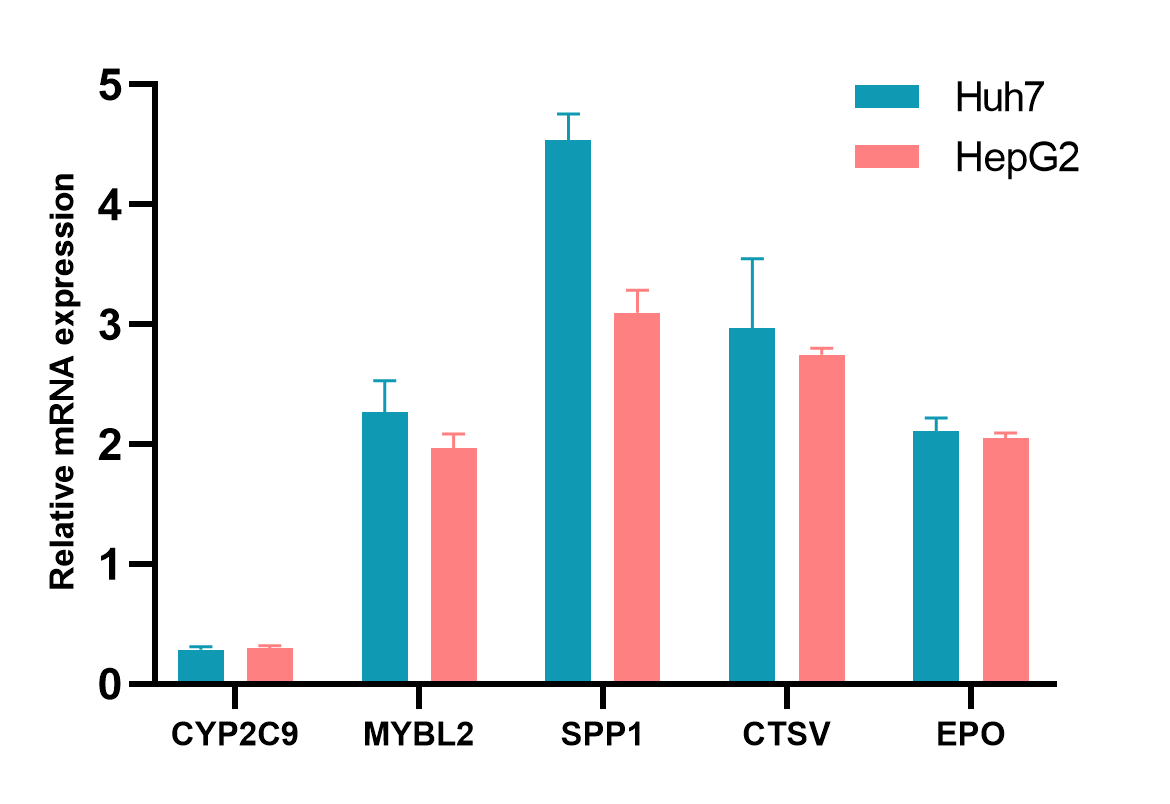

Supplement: Supplemental Information 1 [file peerj-11-14691-s001.zip › Supplementary materials/Figure S6.png]

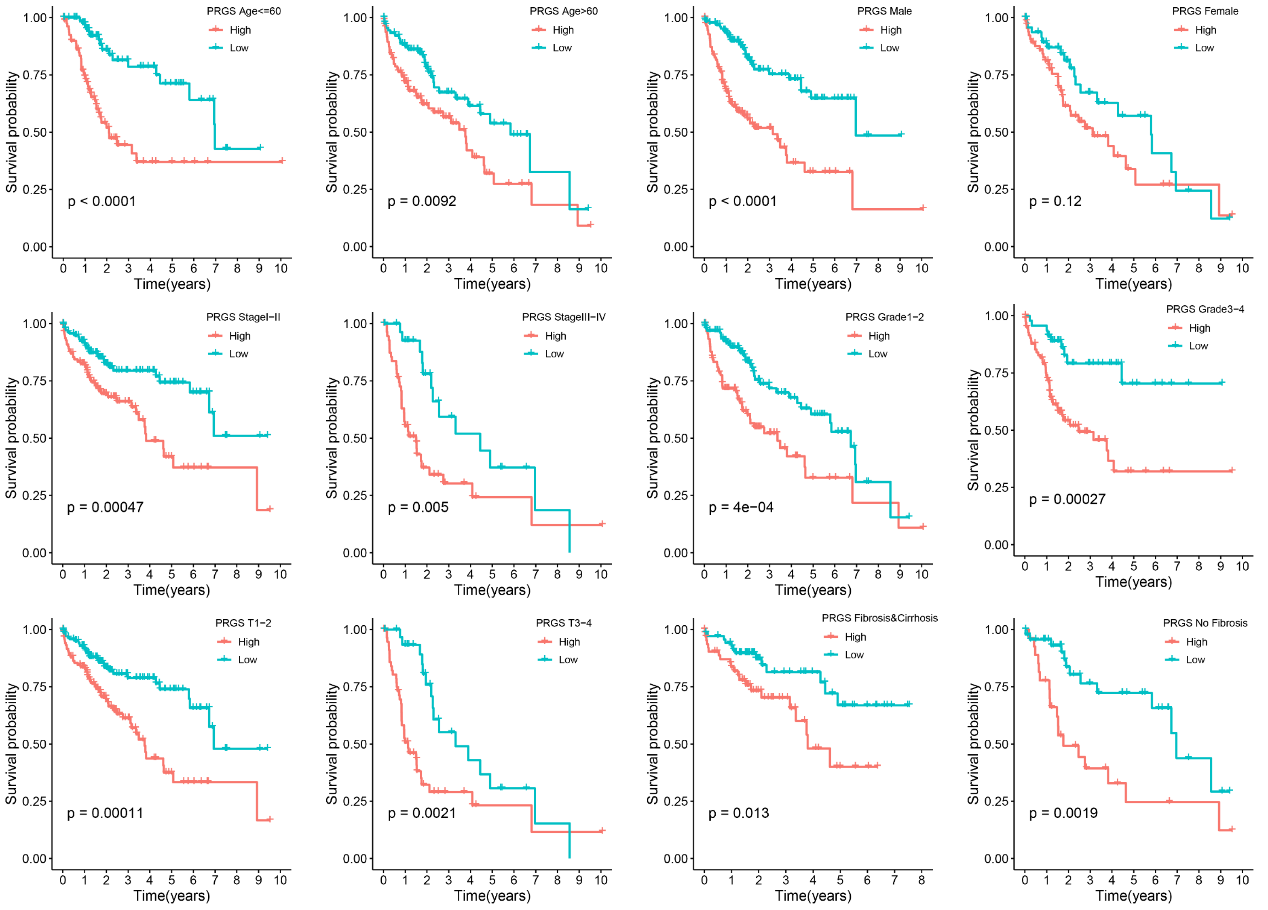

Supplement: Supplemental Information 1 [file peerj-11-14691-s001.zip › Supplementary materials/Figure S7.png]

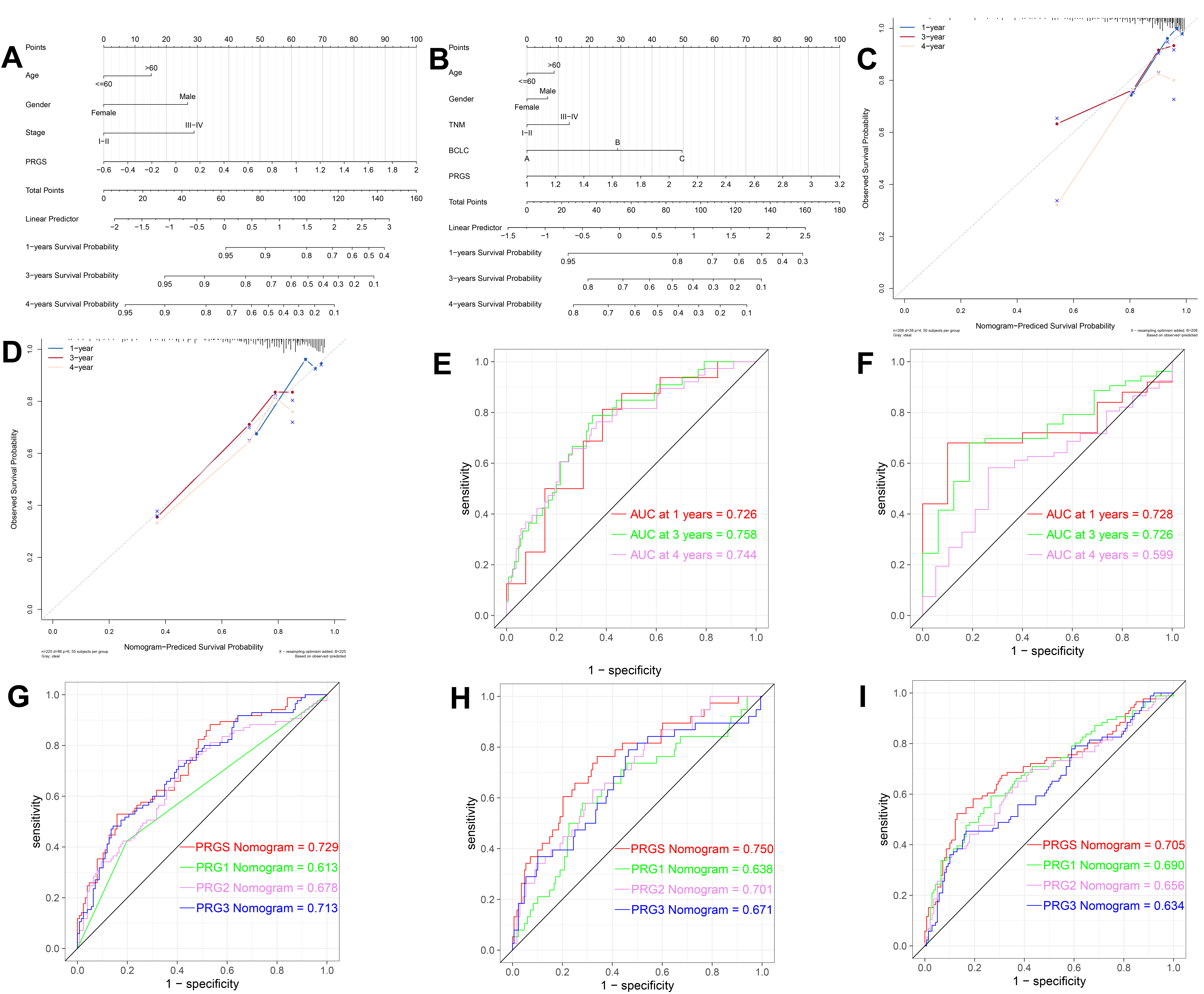

Supplement: Supplemental Information 1 [file peerj-11-14691-s001.zip › Supplementary materials/Figure S8.png]

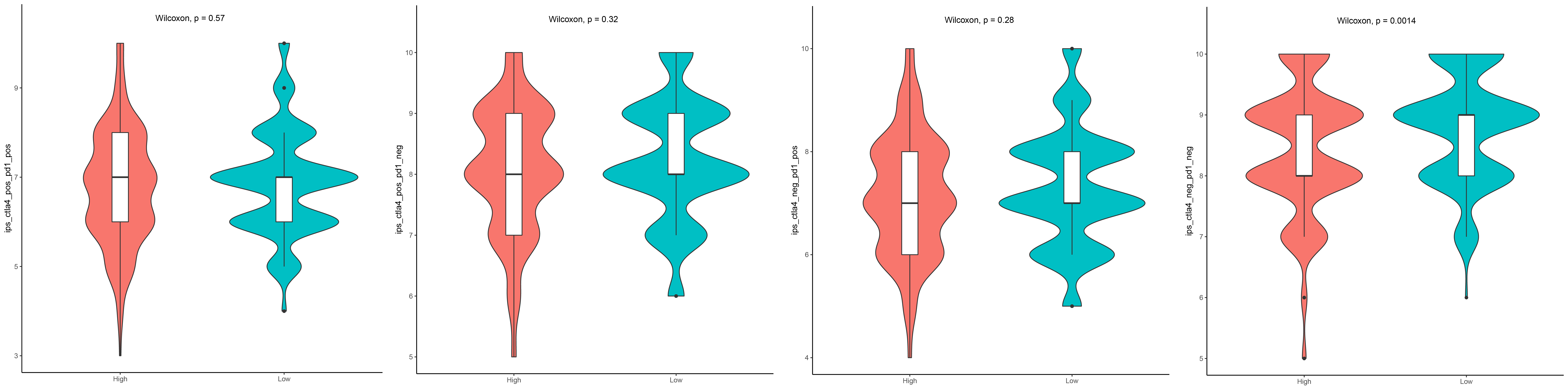

Supplement: Supplemental Information 1 [file peerj-11-14691-s001.zip › Supplementary materials/Figure S9.png]
